# Effectiveness of mHealth Interventions Aimed at Promoting Physical Activity and Reducing Sedentary Behavior on Work-Related Outcomes Among Workers: Systematic Review

**DOI:** 10.2196/80540

**Published:** 2026-05-07

**Authors:** Takako Miki, Michiko Nohara, Nami Homma, Daiki Nagamine, Shinji Yamaguchi, Kyoko Nomura

**Affiliations:** 1 Division of Public Health, Department of Hygiene and Public Health, School of Medicine, Tokyo Women's Medical University, 8-1 Kawada-cho, Shinjuku-ku, Tokyo, 162-8666, Japan, 81 333538111; 2 Department of Environmental Health Science and Public Health, Akita University Graduate School of Medicine, Akita, Japan

**Keywords:** mHealth, physical activity, productivity, sedentary behavior, workers, mobile health

## Abstract

**Background:**

Mobile health (mHealth) technologies have gained popularity and may play a key role in promoting health-related behaviors. However, the impact of mHealth interventions on work-related outcomes remains unclear.

**Objective:**

We aimed to provide an overview of the effectiveness of mHealth interventions designed to encourage physical activity and decrease sedentary behavior on work-related outcomes.

**Methods:**

A search was conducted in MEDLINE, Web of Science Core Collection, the Cochrane Central Register of Controlled Trials and Cochrane Database of Systematic Reviews, and Ichushi-Web for all publications up to September 12, 2025, without language or date restrictions. We included studies which (1) investigated the impact of mHealth interventions promoting physical activity and reducing sedentary behavior on work-related outcomes such as absenteeism, presenteeism, work productivity, work performance, and workability; (2) were designed as a randomized controlled trial or nonrandomized study of interventions; (3) were conducted among workers; and (4) were published as full-text original articles in Japanese or English. Two researchers independently conducted the screening of titles and abstracts, followed by a full-text review to confirm study eligibility and extract the data. We assessed the risk of bias using the Cochrane Collaboration Risk of Bias tool for randomized trials (RoB 2) and the Risk Of Bias In Nonrandomized Studies of Interventions (ROBINS-I). Findings were narratively synthesized.

**Results:**

In total, 4022 records were identified from databases and other sources. After removing duplicates, 3631 studies were screened, and 17 studies (12 randomized controlled trials and 5 nonrandomized studies of interventions) met the inclusion criteria. Of the 17 eligible studies with 18,805 participants, 12 showed a favorable effect direction on ≥1 work-related outcome, but the majority exhibited a high or critical risk of bias, raising concerns about the quality of the research. There was considerable heterogeneity across studies.

**Conclusions:**

Scientific evidence suggests that health promotion efforts that incorporate mHealth components to encourage physical activity and reduce sedentary behavior may have a favorable impact on work-related outcomes. However, the low methodological quality of the studies and their high heterogeneity due to the participant characteristics, intervention content, use of diverse work-related assessment tools, and different follow-up periods make it difficult to draw definitive conclusions about the effectiveness of the interventions. This review is the first to comprehensively synthesize evidence on the impact of mHealth interventions on work-related outcomes, extending prior reviews that primarily focused on traditional workplace-based interventions without mHealth components. This review also contributes to the field by offering guidance for future studies, including the potential value of megastudy designs that evaluate multiple interventions within the same population over the same period on the same outcomes. As a practical implication, these findings suggest that mHealth technologies may serve as a complementary strategy for supporting occupational health and productivity.

## Introduction

Physical inactivity, defined as failure to meet established physical activity guidelines [1], is a major global public health issue in contemporary society with more than 5 million attributed deaths per year [2]. In addition to physical inactivity, sedentary behavior—defined as postures (eg, sitting, reclining, or lying) with characteristic energy expenditure of ≤1.5 metabolic equivalents of task while awake [3]—is also a serious health concern, due to its wide range of adverse health effects, independent of physical activity [4,5]. In particular, working populations are a priority population for these issues, given that a large proportion of their working time is sedentary [6]. Changes in industrial structure have transformed the workplace from one requiring physical labor to one dominated by sedentary work, thereby exposing workers to a range of health risks [7]. Moreover, remote working arrangements have expanded in recent years, and evidence suggests that working from home or teleworking is associated with increased sedentary behavior and reduced physical activity compared to onsite work [8]. These contextual changes underscore the need for scalable and adaptable strategies to reach workers across diverse work settings.

Due to the widespread availability and ubiquity of portable devices, health interventions using mobile health (mHealth) technologies [9] have gained attention for their potential to support lifestyle behaviors [10-12]. mHealth technology, characterized by the apps of portable and wireless systems intended to enhance wellness outcomes, bolster health care delivery, and facilitate medical research [13], offers numerous benefits for promoting health, including accessibility, individualization, real-time feedback, and potential scalability [14]. Several umbrella reviews have reported favorable effects of digital and mHealth interventions on health-related outcomes, such as physical activity and sedentary behavior [10-12]. In addition, a growing body of evidence suggests that higher levels of physical activity and lower levels of sedentary behavior are associated with favorable physical health (eg, musculoskeletal pain [15], cancer, and cardiometabolic diseases [2,4]) as well as mental health (eg, depression) [16,17]. These health conditions are commonly associated with productivity loss [18]. Consequently, strategies targeting these behaviors may in turn contribute to improved work-related outcomes. Consistently, evidence from systematic reviews [19,20] suggests potential beneficial effects of workplace interventions targeting physical activity and/or sedentary behavior on work-related outcomes; however, existing evidence remains limited and precludes drawing definitive conclusions. Moreover, these previous reviews have primarily focused on workplace interventions and have not incorporated mHealth technologies as a key component, thereby limiting the generalizability of their findings. Common barriers to workplace health promotion initiatives include financial constraints and shortages of personnel or expertise [21]. Such challenges can be mitigated through the use of mHealth technologies, which have the potential to provide scalable solutions [14]. To our knowledge, however, systematic reviews or meta-analyses have not assessed the impact of mHealth interventions aimed at promoting physical activity and decreasing sedentary behavior on work-related outcomes.

Given the priority that employers and stakeholders assign to work-related productivity and performance concerns [22], a systematic review of these interventions may provide valuable insights for occupational health management. Considering these issues, the objective of this systematic review is to evaluate the impact of mHealth interventions designed to promote physical activity and reduce sedentary behavior on work-related outcomes, including absenteeism, presenteeism, work productivity, work performance, and workability among workers in comparison with any type of control or comparison condition.

## Methods

### Protocol and Registration

The study protocol was registered in the UMIN Clinical Trials Registry (ID UMIN000052290) and was published elsewhere [23]. This systematic review adhered to the PRISMA (Preferred Reporting Items for Systematic reviews and Meta-Analyses) statement [24]. The PRISMA 2020 checklist is provided in Checklist 1. In addition, the search strategy and reporting follow the PRISMA-S (Preferred Reporting Items for Systematic Reviews and Meta-Analyses literature search extension) [25] to ensure full transparency and reproducibility of search methods (Checklist 2).

### Search Strategy and Databases

As described in the protocol [23], the first systematic search of articles was conducted on September 23, 2023, to retrieve articles published from inception until that date. MEDLINE (via PubMed), Web of Science Core Collection (via Web of Science), the Cochrane Library (including Cochrane Central Register of Controlled Trials [CENTRAL] and Cochrane Database of Systematic Reviews), and Ichushi-Web (Japan Medical Abstracts Society) were searched.

ClinicalTrials.gov and the World Health Organization (WHO) International Clinical Trials Registry Platform (ICTRP) were reviewed via the CENTRAL. Proceedings papers indexed in Web of Science Core Collection and conference proceedings included in CENTRAL were reviewed. Study registries and conference proceedings were reviewed to determine whether full reports of eligible studies had been published. No published or validated search filters were used; the search strategies were developed specifically for this review. The search strategy was adapted from the protocol of a prior scoping review of digital health tools [26] and adjusted appropriately for the current systematic review. The search strategy was a combination of search strings that covered the areas of (1) mHealth technology, (2) physical activity and sedentary behavior, (3) workers (employees), and (4) work-related outcomes. The search strings were first developed within PubMed and then modified for use in the other databases. Consistency across databases was maintained where possible. Terms were searched within multiple fields where available (eg, MeSH [Medical Subject Headings], keywords, title, and abstract). The overall search terms were developed by 2 researchers (TM and KN) and medical librarians at Tokyo Women’s Medical University, who reviewed them to confirm that the required search terms were included and that the overall search approach was appropriate. No date or study design restrictions were applied in the database searches; studies were retrieved from database inception to the search dates. Language restrictions were not applied at the search stage, although studies written in languages other than English and Japanese were excluded during the screening process. In the Japan Medical Abstracts Society database, conference proceedings were excluded to focus on full-text original articles. Snowball searching was performed using the included studies to identify additional relevant literature. The full search strategies for all databases, exactly as executed, are provided in Multimedia Appendix 1. The searches were updated by rerunning the original strategies in each database using the same terms and procedures. All databases were last searched on September 12, 2025. To identify additional studies not captured by electronic database searches, we conducted backward citation searching by hand-screening the reference lists of included studies and relevant reviews. No forward citation searching (eg, using citation indexes or alerts) was performed. No additional information sources or search methods were used beyond the electronic database searches and backward citation searching described above.

### Eligibility Criteria

Population, Intervention, Comparison, and Outcome (PICO) of studies in this systematic review were defined as follows: (P) inclusion of all workers (employees) aged 18 years or older regardless of race and ethnicity; (I) interventions using an mHealth intervention aimed at addressing one or both of the lifestyle factors of physical activity and sedentary behavior as a predominant component; (C) any type of control or comparator condition for comparison (eg, usual care, waitlist group, active control group, or pre- and post-data); and (O) work-associated outcomes (eg, absenteeism, presenteeism, work productivity, work performance, and workability). Health-related behaviors such as physical activity and sedentary behavior were also reported, although they were not definite inclusion criteria. All behavioral measurement types and units were accepted (eg, self-report and steps per day, and daily or weekly moderate-to-vigorous physical activity time).

### Inclusion Criteria

Studies were included based on the following criteria:

1. Studies targeting all workers without consideration of employment status (full-time or part-time), job type, or shift type. No restrictions were placed on current diseases or risk factors.
2. Studies including mHealth interventions encouraging physical activity and decreasing sedentary behavior, for disease prevention and treatment or management of health conditions (eg, back pain or diabetes) aimed at workers. Workers with these conditions were included due to the substantial burden of lost work productivity involved [18]. Intervention setting was limited to nonclinical settings (eg, workplace, home, or outdoors).
3. Studies including mHealth interventions via mobile devices: mobile phones, smartphones, personal digital assistants, tablets, wearable activity monitors or trackers, mobile apps, SMS, and other wireless devices. Consistent with a previous systematic review [27], standard pedometers (unable to transmit data to consumer interface) were not considered mHealth components due to the lack of an electronic communication function with mobile interfaces and the internet [28].
4. Studies using mHealth technologies to promote physical activity or reduce sedentary behavior alone or with other behaviors (eg, diet). Multicomponent strategies were included where mHealth technology was one of several intervention components (eg, face-to-face counseling), as a prior review reported many mHealth interventions for physical activity and sedentary behavior were delivered as part of multicomponent interventions [29]. Inclusion of multicomponent strategies broadened the range of eligible studies and review scope. Although multicomponent interventions may increase heterogeneity and make determining whether effects are attributable to mHealth or other components difficult, mHealth interventions alone are often associated with low adherence and high attrition rates [30,31]. Given that the National Institute for Health and Care Excellence (NICE) committee recommended considering digital and mHealth interventions alongside existing services [32], multicomponent interventions were included.
5. Studies with any type of control or comparator condition for comparison.
6. Studies assessing work-related outcomes: absenteeism, presenteeism, work productivity, work performance, and workability.
7. Studies published as full-text original articles.
8. Studies conducted as randomized controlled trials (RCTs) or nonrandomized studies of interventions (NRSIs) with predefined control or comparator group (eg, nonrandomized controlled trials, cohort studies, and pre-post studies) [33]. NRSI design was included based on a scientific overview of occupational health interventions, encouraging researchers to include RCT alternatives when conducting systematic reviews [34]. This methodology provides broad evidence to guide real-world implementation where RCT would be difficult or inappropriate. Current guidance considers NRSI evidence prominent in decision-making [33]. Pilot and feasibility trials were included if they met the inclusion criteria. Quantitative and mixed methods studies were included to ensure all relevant quantitative results were obtained; for mixed methods, only quantitative results were extracted.
9. Studies published in Japanese or English. In addition to English studies, Japanese studies were also included to capture domestic research trends. No translation procedures were required for these languages in this review. To minimize language bias, no language restrictions were applied during literature searching, but studies were limited to Japanese and English during study selection.

### Exclusion Criteria

Studies were excluded based on the following criteria:

1. Studies reporting results from samples mixing workers with other groups (eg, full-time students or retired older adults), unless results are reported independently for each group.
2. Non–full-text original articles (eg, editorials, reviews, protocols, letters to the editor, published conference abstracts, dissertations, books, and case reports or case series). Conference proceedings, trial registry records, and protocol papers identified during screening were not included as eligible studies in this review. However, their details were reviewed to confirm whether any original articles relevant to this review had been published.
3. Studies having an inappropriate design (eg, without a control or comparator group, accuracy or validation studies, descriptive articles, exclusively qualitative research, and mixed methods with quantitative results unsuitable for inclusion).

### Study Selection Process

Records identified from the searches were managed in Microsoft Excel. Duplicate records were identified and removed manually by TM through comparison of titles and bibliographic information (eg, DOI). After duplicate removal, 2 researchers (TM and either NH, DN, or SY) independently performed initial screening by reading the titles and abstracts, then obtained the full text of retained studies for independent full-text screening. At the first screening, the kappa statistic of each pair of researchers was 0.77. The corresponding value at the full-text review stage was 0.76. Inconsistent assessments were resolved through discussion among the authors. The reasons for excluding particular studies are recorded in Multimedia Appendix 2. During the screening and selection process, TM contacted the corresponding authors of potentially eligible studies to clarify unclear information, request additional data when necessary, and confirm eligibility for inclusion in the review.

### Data Extraction

Information from each of the included studies was extracted by 2 researchers (TM and either NH, DN, or SY) using a standardized data extraction form. The data to be extracted were predetermined, and their details were described in the protocol [23]. Briefly, information included author, year of publication, country, study design, participant information, intervention details, control or comparison group, work-related outcomes, summary results for each study, adverse events, adherence to the intervention, and source of funding. After extraction, information was confirmed through discussion among all authors until consensus in data collection was reached. If the studies did not include this information and/or contained unclear information, TM contacted the corresponding authors for clarification. All data extracted from each individual study included in the results of this systematic review are provided in a data extraction table (extracted data in Multimedia Appendix 3).

### Quality Assessment

Bias risk for RCTs was assessed using the Revised Cochrane Collaboration Risk-of-Bias tool (RoB 2) [35], which assesses five risk domains, namely (1) randomization process; (2) deviations from the intended intervention; (3) missing outcome data; (4) outcome measurement; and (5) selection of reported results. For cluster RCTs, we assessed the 5 risk domains above and an additional domain for cluster RCTs [35]. Risk of bias (RoB) for NRSIs was assessed using the Cochrane ROBINS-I (Risk Of Bias In Nonrandomized Studies of Interventions) tool [36], which assesses bias based on (1) confounding factors, (2) participant selection, (3) classification of interventions, (4) deviations from intended interventions, (5) missing outcome data, (6) outcome measurement, and (7) selection of reported results. The RoB in each included study was independently evaluated by researchers (NH, DN, and SY) and confirmed by TM. Interrater agreement for RoB assessments was 73.9% (17/23 outcomes). Disagreements were resolved through discussion until a consensus was reached.

### Data Synthesis

Due to substantial heterogeneity among the included studies in participant characteristics, intervention content, work-related outcome measures, and follow-up periods, quantitative synthesis through meta-analysis could not be conducted. Instead, a narrative synthesis approach was used. The certainty in the body of evidence (eg, using the Grading of Recommendations Assessment, Development and Evaluation [GRADE] system) was not formally assessed because the review adopted a narrative synthesis approach. The substantial heterogeneity among the included studies precluded the meaningful application of quantitative certainty grading systems. A narrative synthesis of the findings was conducted in line with the Synthesis Without Meta-Analysis (SWiM) guideline (Multimedia Appendix 4) [37]. We narratively synthesized the direction of results of individual studies using the vote-counting system [38] and visualized them using the modified effect direction plot [38,39]. The revised effect direction plot provides a comprehensive summary of the impact of mHealth interventions on work-related outcomes and health-related outcomes (Table S1 in Multimedia Appendix 3). Studies were ordered first by intervention category based on mHealth tools (mobile apps, web-based components, combinations of mobile apps and wearable activity monitors or trackers) and then chronologically by year of publication within each category. In addition, studies were further categorized based on the primary behavioral target of the intervention (physical activity, sedentary behavior, or both) to explore whether intervention focus influenced work-related outcomes (Table S2 in Multimedia Appendix 3). Heterogeneity was explored by comparing the direction of effects across these subgroups. Patterns of consistency or variation in effect direction were examined narratively and visually using the modified effect direction plot. All eligible studies, regardless of RoB or study design, contributed to the vote-counting synthesis. However, the overall conclusions were based on a comprehensive assessment, including methodological quality (RoB) and heterogeneity across studies. Following recommendations in the Cochrane Handbook for Systematic Reviews of Interventions, vote counting based on direction of effect was used for the synthesis of results [38,40]. In line with the guidance [38,40], we did not take statistical significance or magnitude of effect into account within this synthesis. We did not consider the statistical significance of effects as attention to this feature may lead to the exclusion of underpowered studies [40]. Owing to the highly heterogeneous study characteristics (such as the lack of a consistent effect measure), we relied on the direction of effects as a standardized binary metric. Effect estimates were categorized as favorable (favoring the intervention group) or unfavorable (favoring the control or comparison group) based on the direction of effect. For work-related outcomes, increases in work productivity, work performance, and workability were considered favorable, whereas decreases in absenteeism and presenteeism were also considered favorable. For health-related outcomes, increases in physical activity were considered favorable, whereas decreases in sedentary behavior were considered favorable. To implement vote counting based on the direction of effect, we followed the study-level step-by-step procedures detailed by Boon and Thomson [38]:

1. We grouped studies for synthesis according to predefined categories of work-related outcomes (ie, absenteeism, presenteeism, work productivity, work performance, and work ability) and health-related outcomes (ie, physical activity and sedentary behavior). This grouping was chosen because these outcomes represent conceptually distinct domains and are commonly evaluated separately in prior occupational health research [19]. No changes were made to the categorization of these outcomes from those prespecified in the study protocol [23].
2. We counted favorable or unfavorable effect estimates for each type of work-related outcomes and health-related outcomes.
3. We determined the overall effect direction for each domain using the following algorithm. If multiple outcomes within a study reported effects in the same direction, that direction was assigned to the domain. If the direction of effect varied across outcomes, the domain was assigned the direction reported by ≥70% (ie, a clear majority) of outcomes. If fewer than 70% of outcomes showed a consistent direction of effect, or if effect estimates were identical between the intervention and control or comparison groups, or were not reported, the domain was classified as having no clear effect direction. Furthermore, we systematically tabulated the key characteristics and results of the included studies to facilitate comparison and interpretation. Data extracted from the included studies were ordered chronologically by publication date (extracted data in Multimedia Appendix 3). Individual study findings were reported using the effect measures as presented in the original articles, without reexpressing them using a different effect measure.

## Results

### Overview

**Figure 1.**
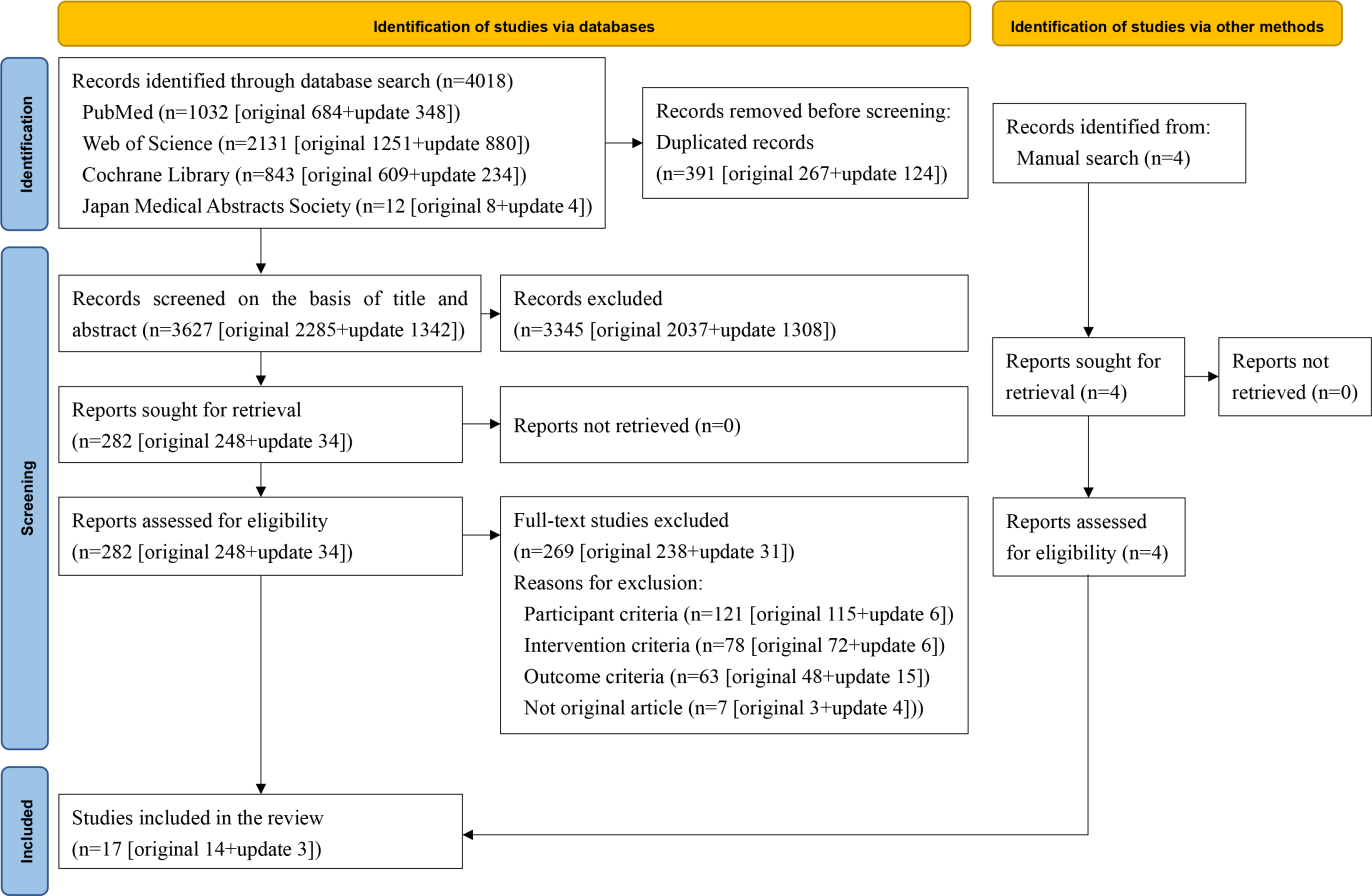
PRISMA (Preferred Reporting Items for Systematic reviews and Meta-Analyses) 2020 diagram.

The PRISMA 2020 flow diagram [24] of the study selection process is shown in Figure 1. The search of the 4 databases identified a total of 4018 records. After removing 391 duplicates, 3627 records were included in the screening on the basis of title and abstract, during which 3345 records were excluded, and 282 records were selected to proceed to full-text screening. Subsequently, we excluded 269 studies that did not meet the eligibility criteria for participants (n=121), intervention (n=78), outcomes (n=63), or publication type (n=7). Four additional studies identified through sources other than the database search met the eligibility criteria. Finally, 17 studies [41-57] were included in this review.

### Study Characteristics

An overview of the study characteristics is given in Table 1. An extended version of the table is available in Multimedia Appendix 3 (extracted data). Out of the 17 included studies [41-57], 12 (70.6%) [41-52] were RCTs (including 4 cluster RCTs [49-52]) and 5 (29.4%) used NRSIs [53-57]. These NRSIs consisted of 3 retrospective cohort studies [53-55], 1 prospective cohort study [57], and 1 single-group, pre-post, mixed methods pilot study [56]. All included studies except one [44] (16/17, 94.1%) were conducted from 2016 onward [41-43,45-57]. Out of the 17 included studies [41-57], 7 (41.2%) were conducted in European countries: 2 each in England [52,56], Spain [46,51], and Finland [44,48], and 1 in Germany [42]. Of the remaining studies, 5 (29.4%) were conducted in North America: 3 in the United States [54,55,57], 2 in Canada [43,45], and 3 (17.6%) were conducted in Asia: 2 in Japan [41,53] and 1 in Thailand [49]. Other countries included Australia [50] and Brazil [47].

**Table 1.** The characteristics of the included studies (N=17).

Characteristics	Values
Study design, n (%)	
RCTs^a^	12 (70.6)
NRSIs^b^	5 (29.4)
Publication year, n (%)	
<2016	1 (5.9)
≥2016	16 (94.1)
Country, n (%)	
Europe	7 (41.2)
North America	5 (29.4)
Asia	3 (17.6)
Others	2 (11.8)
Total sample size, n (%)	
<100	4 (23.5)
101‐300	8 (47.1)
301‐1000	2 (11.8)
>1000	3 (17.6)
Target participants, n (%)	
Apparently healthy workers	10 (58.8)
Workers with certain health conditions	7 (41.2)
Percentage of females, n (%)	
<60%	9 (52.9)
≥60%	8 (47.1)
Mean age categories, n (%)	
<40 years old	4 (23.5)
≥40 years to <50 years old	11 (64.7)
≥50 years old	2 (11.8)
Duration of intervention, n (%)	
<12 weeks	3 (17.6)
≥12 weeks to <12 months	9 (52.9)
12 months	3 (17.6)
Not clearly described	2 (11.8)
Intervention focus, n (%)	
Targeted physical activity	11 (64.7)
Targeted sedentary behavior	3 (17.6)
Targeted both physical activity and sedentary behavior	3 (17.6)
Type of mHealth^c^ tools, n (%)	
Mobile apps	6 (35.3)
Combinations of mobile apps and wearable activity monitors or trackers	8 (47.1)
Web-based components	3 (17.6)
Intervention strategies, n (%)	
mHealth-only	4 (23.5)
Blended	13 (76.5)
Adherence to the intervention, n (%)	
Described	10 (58.8)
Not described	7 (41.2)
Control or comparison for work-related outcomes, n (%)	
Passive control	8 (47.1)
Active control	4 (23.5)
Pre-post comparison	5 (29.4)
Work-related outcomes, n	
Absenteeism	5
Presenteeism	2
Work productivity	7
Work performance	4
Workability	3
Health-related outcomes, n	
Physical activity	10
Sedentary behavior	7
Not described	7
Adverse event, n (%)	
Described	8 (47.1)
Not described	9 (52.9)

a RCT: randomized controlled trial.

b NRSI: nonrandomized studies of intervention.

c mHealth: mobile health.

### Participant Characteristics

Total sample sizes ranged from <100 (4/17, 23.5%) [41,43,46,47], 101‐300 (8/17, 47.1%) [45,48-52,55,56], 301‐1000 (2/17, 11.8%) [42,44], and >1000 (3/17, 17.6%) [53,54,57]. Ten studies (10/17, 58.8%) focused on apparently healthy workers; the majority recruited employees who were identified as office workers [43,44,49-52], while one study each was performed in health care workers [45], police officers and staff [56], workers registered with an internet survey company [53], and military workers [48]. The other 7 studies (7/17, 41.2%) targeted workers with certain health conditions, namely metabolic syndrome [42]; off-work and undergoing rehabilitation for a wrist, hand, and/or finger injury [46]; chronic low back pain [41,47]; knee or low back pain [54]; prediabetes [55]; and pelvic floor disorders [57]. Out of the 17 included studies [41-57], 8 studies (47.1%) had a higher proportion of female participants (≥60%) [43-45,49,51,52,55,57]. The mean age of participants in the included studies ranged from 38.6 (SD 10.4) to 54.3 (SD 6.5) years. Most included studies (11/17, 64.7%) [41-46,48,51-54] reported mean participant ages between 40 and 49 years.

### Intervention Characteristics

#### Intervention Duration and Target Behavior

The duration of the interventions ranged from 6 weeks to 12 months, with most studies reporting durations between 12 weeks and less than 12 months (9/17, 52.9%) [41,42,45,48,49,51,54-56]. Of the 17 studies reviewed [41-57], 11 (64.7%) primarily focused on promoting physical activity as their main intervention strategy [41,42,44-46,48,53-55,57], 3 (17.6%) focused on reducing sedentary behavior [43,50,52], and 3 (17.6%) targeted both physical activity and sedentary behavior [49,51,56].

#### Type of mHealth Tools

The main mHealth tools were combinations of mobile apps and wearable activity monitors or trackers (8/17, 47.1%) [42,48-50,54-57], followed by mobile apps (6/17, 35.3%) [41,45-47,52,53]. The remaining 3 studies (3/17, 17.6%) used web-based interventions [43,44,51].

#### Intervention Strategies

When interventions were defined as either mHealth-only or blended strategies (mHealth combined with other components such as personal counseling), 4 studies [45,47,51,53] were classified as mHealth-only and the remaining 13 [41-44,46,48-50,52,54-57] as blended interventions. The majority of studies (13/17, 76.5%) included components other than mHealth technology in their interventions.

#### Adherence

With regard to adherence to the intervention (ie, the extent to which users followed the program as designed), 10 studies (10/17, 58.8%) [41,42,44,45,49,50,52,54,56,57] reported information on adherence whereas the remaining 7 (7/17, 41.2%) [43,46-48,51,53,55] did not provide detailed information on adherence. In the absence of detailed information on adherence reported within the publication, additional data [58,59] were obtained through direct correspondence with the study authors. Due to the differences in defining and measuring adherence, as well as variations in the duration and content of the intervention across studies, comparing adherence rates between included studies was challenging. Overall, adherence to the intervention tended to decrease over time. Among mHealth devices, however, Fitbit devices tended to maintain high adherence rates [49,56,58]. One study reported high Fitbit adherence, with a mean wear time of 6.5 (SD 1.1) days per week and 21.4 (SD 4.1) hours per day at 8-month follow-up [56], whereas another study reported typical wear times of 10‐15 hours per day at baseline, decreasing slightly to 8‐14 hours per day after 2‐3 months of the intervention (SDs for both time points were not reported in the original study) [49,58].

### Control or Comparison Characteristics

We defined passive controls as those comprising no intervention, usual practice, or a waitlist, and active controls as comprising components intended to induce changes in one or more outcomes. Of the 17 included studies [41-57], 8 (47.1%) used passive controls [41-45,48,52,53], 4 (23.5%) used active controls [46,47,49,51], and 5 (29.4%) used pre-post comparison within the same group (pre-post design) for target work-related outcomes [50,54-57].

### Work-Related Outcomes

The included studies were evaluated for work-related outcomes such as absenteeism, presenteeism, work productivity, work performance, and workability. The most common work-related outcome was work productivity in 7 studies [41,44,49,51,54,56,57], followed in order by absenteeism in 5 [44-46,52,55], work performance in 4 [43,50,52,53], workability in 3 [42,47,48], and presenteeism in 2 [51,52]. Only 4 work-related outcomes were assessed as primary outcomes [41,44,46]; the majority of work-related outcomes were measured as secondary [41-43,45,47,48,50-57] or tertiary outcomes [49]. No overall certainty assessment for each work-related outcome was conducted due to the narrative synthesis approach.

### Work Productivity Outcomes

Results summarizing the impact of mHealth interventions on work-related outcomes and health-related outcomes are presented in Table S1 in Multimedia Appendix 3. Out of the 7 studies [41,44,49,51,54,56,57], 5 studies (71.4%) [41,49,51,54,57] reported favorable effect directions with work productivity outcomes in the intervention group compared with the control or comparison groups. Of the 5 studies that showed favorable effect directions [41,49,51,54,57], 3 [41,49,51] were RCTs (including 2 cluster RCTs [49,51]) and 2 [54,57] were NRSIs. Among these studies, 3 targeted physical activity [41,54,57], whereas 2 targeted both physical activity and sedentary behavior [49,51]. Regarding the mHealth components, one study [41] used mobile apps as part of the intervention, whereas another [51] conducted a web-based mHealth intervention. The remaining 3 studies [49,54,57] implemented mHealth tools combining mobile apps with wearable activity monitors or activity trackers. The remaining 2 studies (2/7, 28.6%) [44,56] showed no clear direction of effect for this outcome.

#### Impact of mHealth Interventions Primarily Targeting Physical Activity on Work Productivity Outcomes

Results summarizing the impact of mHealth interventions on work-related outcomes and health-related outcomes by targeted intervention types are shown in Table S2 in Multimedia Appendix 3. Out of the 4 studies [41,44,54,57] primarily aimed at increasing physical activity, 3 (75%) [41,54,57] reported favorable effect directions with work productivity outcomes in the intervention group compared with the control or comparison groups. Of the 3 studies that showed favorable effect directions [41,54,57], one [41] was an RCT and the remaining 2 were NRSIs [54,57]. In detail, a Japanese study with an RCT design [41] explored the effects of a 12-week patient education and strengthening exercise program delivered using a mobile messaging app on work productivity among patients receiving pharmacological treatment for chronic low back pain. The conventional group received only routine medical care. The between-group differences for work productivity outcomes favored the intervention group but did not reach statistical significance. Regarding the NRSI studies, the retrospective cohort study [54] among workers with either knee or low back pain in the United States evaluated the efficacy of a remote digital care program on self-reported work productivity as assessed by the Work Productivity and Activity Impairment (WPAI) questionnaire. The digital care program was delivered through a mobile app that incorporated education, sensor-guided exercise therapy, and behavioral health support given 1-on-1 by a remote health coach. The results demonstrated statistically significant improvements in WPAI-assessed work productivity from baseline to the 12-week follow-up. Additionally, in another NRSI study using a prospective cohort design [57], a digital pelvic program was conducted for postmenopausal women with pelvic floor disorders. This program combined pelvic floor muscle training (PFMT) and education, using a combination of mobile apps and wearable trackers. During the PFMT sessions, pelvic floor muscle activity was assessed with a tracker, and real-time feedback was provided through the mobile app. Work productivity impairment was reduced by around half at the intervention end (mean difference −15.08, 95% CI −17.52 to −12.64; *P*<.001). In contrast, an RCT with employees at a Finnish insurance company [44] showed no clear between-group difference in work productivity, with adjusted mean differences in the quantity and quality of work index of 1.3 (−2.0 to 4.7) and −1.1 (−4.9 to 2.8) at 6 and 12 months, respectively. This study used web-based components [44] as an mHealth technology in the intervention. Participants in the intervention group monitored their daily physical activity, set goals, accessed a web-based tracking service, and received counseling via telephone or web messages over a 12-month period. Meanwhile, those in the control group were informed of the results of a fitness test and an information leaflet on physical activity with continued occupational health care as usual.

#### Impact of mHealth Interventions Primarily Targeting Sedentary Behavior on Work Productivity Outcomes

No included studies examined the impact of mHealth interventions mainly targeting sedentary behavior on work productivity outcomes.

#### Impact of mHealth Interventions Primarily Targeting Both Physical Activity and Sedentary Behavior on Work Productivity Outcomes

Out of the 3 studies [49,51,56] targeted both physical activity and sedentary behavior, 2 (66.7%) [49,51] reported favorable effect directions with work productivity outcomes in the intervention group compared with the control group. A cluster RCT among employees at 6 Spanish university campuses [51] examined a 19-week workplace web-based intervention that aimed to encourage office employees to increasingly “sit less and move more” during workdays. In this study, loss of performance, as assessed by the Work Limitation Questionnaire (WLQ), showed a statistically significant increase in both the intervention group (workplace web-based intervention) and active control group (pedometer, paper diary, and self-reported sitting time) across all study time points. However, after baseline, work performance was consistently better in the intervention group compared with the active comparison group. Likewise, in another cluster RCT [49] among office workers in Thailand, the intervention showed a slightly favorable effect direction for work productivity, as assessed by the WPAI. The adjusted between-group difference for percentage reduced work productivity was *β*=−.37 (95% CI −9.20 to 8.47), although the difference was not statistically significant. This study investigated the impact of a 6-month multicomponent short-break intervention aimed at decreasing sitting time and increasing physical activity, which included real-time feedback on activities that could be viewed via a smartwatch or using the Fitbit smartphone app (Fitbit Inc). Additionally, both this intervention group and an active control group received an information booklet on the benefits and consequences of physical activity and 7 simple exercises for promoting an active lifestyle in the office environment. In contrast, a single-group, pre-post mixed methods pilot study among police officers and staff in the United Kingdom [56] evaluated a 12-week mHealth intervention (Fitbit activity monitor and the “Bupa Boost” smartphone app; developed by Bupa). The study reported no statistically significant changes in perceived productivity, as assessed by the World Health Organization Health and Work Performance Questionnaire (WHO-HPQ), from baseline to week 6 (midintervention) or from baseline to week 12 (postintervention). Since the effect estimates of these changes were not provided, the effect direction was considered unclear.

### Absenteeism Outcomes

Out of the 5 included studies [44-46,52,55] that evaluated absenteeism outcomes, 3 (60%)—2 RCTs [45,46] and a retrospective cohort study [55] targeting physical activity—demonstrated favorable effect directions with absenteeism outcomes in the intervention group compared with the control or comparison groups. Of the 3 studies that showed favorable effect directions [45,46,55], 2 used mobile apps as the mHealth tools for the intervention [45,46], whereas one used mobile apps and wearable activity trackers as mHealth technologies as part of the intervention [55]. The remaining 2 studies (2/5, 40%) [44,52] showed no clear direction of effect for this outcome.

#### Impact of mHealth Interventions Primarily Targeting Physical Activity on Absenteeism Outcomes

Out of the 4 studies [44-46,55] primarily targeting physical activity, 3 (75%) [45,46,55] reported favorable effect directions for absenteeism outcomes in the intervention group compared with the control or comparison groups. In one RCT [45] performed among health care workers in Canada, participants assigned to the exercise condition were instructed to install the Down Dog suite of apps (Yoga Buddhi Co), which includes body weight interval training, yoga, running, and barre. They were asked to undertake four 20-minute sessions each week (totaling 80 min/wk) over a 12-week period, either at home or at any convenient location. In contrast, participants in the waitlist control condition were instructed to continue their current level of physical activity. The results showed that participants in the exercise intervention group had fewer weeks with reported sick days than those in the control condition. In another RCT [46] conducted among workers with bone and soft-tissue injuries affecting the wrist, hand, or fingers in Spain, participants in the intervention group received a feedback-guided home exercise program delivered via a tablet-based app. Participants receiving the intervention experienced a hastened return to work compared with those in the control group who received the conventional paper-based program (mean between-group difference –18 d, 95% CI –33 to –3). Further, a retrospective cohort study [55] provided prediabetic adults with a novel personalized digital diabetes prevention program through a smartphone app, which incorporated remote monitoring, interactive mobile computing, behavior tracking tools (Fitbit Flex2 [Fitbit Inc]), an evidence-based curriculum, online peer support, and health coaching to prevent or delay type 2 diabetes onset. Results showed that work absenteeism decreased by nearly half a day per month, with a statistically significant difference in WHO-HPQ–measured absenteeism scores between baseline (mean 0.9, SD 1.2 d) and 4 months (mean 0.5, SD 1.1 d; *P*<.001). In contrast, one RCT [44] described in detail in the section on work productivity did not report a clear difference between the web-based intervention and control groups in accumulated sickness absence days during 12 months (adjusted mean difference 0.0 d; 95% CI −1.2 to 0.9).

#### Impact of mHealth Interventions Primarily Targeting Sedentary Behavior on Absenteeism Outcomes

In a cluster RCT performed among desk-based workers [52], participants in the intervention group were provided with a height-adjustable workstation, attended a short seminar and supporting leaflet, received workstation instructions as well as sitting and standing targets, posters, a booklet on action planning and goal-setting, feedback on physical and sitting activity, a self-monitoring prompting tool (the DARMA cushion, which can sync data with a mobile app using Bluetooth), and coaching sessions (month 1 and quarterly), while participants in the control group received the results of health measures (eg, weight and blood pressure) and continued with their usual practice for the 12-month study period. Based on sickness absence data obtained from self-reports and organizational records, no clear difference was observed between the intervention and passive control (usual practice) groups. For self-reported data, the adjusted mean differences were 0.02 (95% CI −0.01 to 0.05) at 3 months, −0.02 (95% CI −0.05 to 0.02) at 6 months, and −0.02 (95% CI −0.10 to 0.06) at 12 months. Organizational records also showed adjusted mean differences of 1.32 days missed (95% CI −9.99 to 12.63) and 0.24 episodes (95% CI −0.35 to 0.82) from pre-intervention to 12 months.

#### Impact of mHealth Interventions Primarily Targeting Both Physical Activity and Sedentary Behavior on Absenteeism Outcomes

No included studies examined the impact of mHealth interventions mainly targeting both physical activity and sedentary behavior on absenteeism outcomes.

### Work Performance Outcomes

Out of the 4 studies [43,50,52,53], 2 studies (50%)—an RCT [43] and a cluster RCT [52] targeting sedentary behavior—reported favorable effect directions on work performance outcomes in the intervention group compared with the control group. These studies used either mobile apps [52] or web-based components [43] as mHealth technologies in their interventions. However, one retrospective cohort study (25%) [53] that used a mobile app showed a slightly unfavorable effect direction for work performance outcomes. The remaining study (1/4, 25%) [50] showed no clear direction of effect for this outcome.

#### Impact of mHealth Interventions Primarily Targeting Physical Activity on Work Performance Outcomes

A retrospective cohort study [53] conducted among full-time workers registered with an internet survey company in Japan investigated the association between Pokémon GO (Niantic) use and work performance measured using the WHO-HPQ. Pokémon GO is a smartphone game app that uses location-based data and augmented reality to allow players to find, catch, and train Pokémon in real-world settings, as well as collect in-game items. The app also features “Pokémon eggs,” which can only be hatched after walking a certain distance. As a result, it encourages players to move around. The study indicated an unfavorable effect direction, with job performance decreasing to a slightly greater extent among Pokémon GO players than nonplayers, although the difference was not statistically significant (group × time *P*_interaction_=.38; Cohen *d*=−0.07; 95% CI −0.21 to 0.06).

#### Impact of mHealth Interventions Primarily Targeting Sedentary Behavior on Work Performance Outcomes

Out of the 3 studies targeting sedentary behavior [43,50,52], 2 studies (66.7%)—1 RCT [43] and 1 cluster RCT [52]—showed favorable effect directions on work performance in the intervention group compared with the control group. In detail, the cluster RCT [52], which is described in detail in the absenteeism section, found group differences in favor of the intervention group compared with control for work performance at 3 months (*β* coefficient=0.24, 95% CI −0.12 to 0.61), 6 months (*β* coefficient=0.41, 95% CI 0.05-0.77), and 12 months (*β* coefficient=0.53, 95% CI 0.20-0.86). In addition, a secondary analysis of an RCT among office workers in Canada [43] examined the effect of a 6-week theory-based planning and web-based text message intervention targeting workplace sitting duration. The intervention group reported greater improvements in work performance compared to the control group, although the interaction effect was not statistically significant (*F* ₁,₅₈=1.67; *P*=.20, $\eta_{p}^{²}$=.03). In contrast, in a cluster RCT among desk-based office workers in Australia [50], the effect direction on work performance assessed using a 9-item self-rated scale was considered unclear because no group comparisons were conducted between the organizational support alone group (Group ORG) and the organizational support combined with the LUMOback activity tracker group (Group ORG + Tracker). Neither group showed significant changes in job performance at 3 or 12 months compared with baseline.

#### Impact of mHealth Interventions Primarily Targeting Both Physical Activity and Sedentary Behavior on Work Performance Outcomes

No included studies examined the impact of mHealth interventions mainly targeting both physical activity and sedentary behavior on work performance outcomes.

### Workability Outcomes

Out of the 3 RCTs targeting physical activity [42,47,48], 2 studies (66.7%) [42,48] reported favorable effect directions on workability outcomes in the intervention group compared with the control group. These 2 studies used mobile apps and wearable activity monitors as mHealth technologies as part of the intervention [42,48]. The remaining study (1/3, 33.3%) [47] showed no clear direction of effect for this outcome.

#### Impact of mHealth Interventions Primarily Targeting Physical Activity on Workability Outcomes

Of the 3 studies [42,47,48] targeting physical activity on workability outcomes, 2 [42,48] reported favorable effect directions in the intervention group compared with the control group. In one RCT [42], participants in the exercise group were provided with individual recommendations for exercise at in-person meetings and using a smartphone app, Rebirth Active (d.velop AG; with the Garmin activity monitor), with the aim of doing 150 minutes of physical activity per week. This study observed that workability improved at 6 months of the exercise intervention compared to the waitlist control group. Total Work Ability Index score increased in the exercise group but not in the control group (waitlist), with a statistically significant difference of 1.50 points (95% CI 0.75-2.25; *P*<.001) between groups. In another RCT [48] performed among military employees in Finland, participants in the intervention group were provided with an interactive accelerometer (ExSed Movesense and Suunto) linked to a mobile app (ExSed [UKK, Terveyspalvelut Oy]) and a cloud service, along with telephone counseling and encouragement to exercise. The control group continued their usual physical activity without the accelerometer or feedback. Workability was measured on a 0‐10 scale comparing current workability with the highest level during one’s career, with higher scores indicating better workability. The study reported that workability scores decreased in both the intervention and control groups during the 1-year follow-up, but the decline was smaller in the intervention group, with no statistically significant group × time interaction (*t*=1.020; *P*=.31). In contrast, in the remaining RCT study [47] conducted among public safety workers with chronic low back pain, no clear effect directions on workability were observed because between-group comparisons were not performed.

#### Impact of mHealth Interventions Primarily Targeting Sedentary Behavior on Workability Outcomes

No included studies examined the impact of mHealth interventions mainly targeting sedentary behavior on workability outcomes.

#### Impact of mHealth Interventions Primarily Targeting Both Physical Activity and Sedentary Behavior on Workability Outcomes

No included studies examined the impact of mHealth interventions mainly targeting physical activity and sedentary behavior on workability outcomes.

### Presenteeism Outcomes

Both 2 included cluster RCTs (2/2, 100%) [51,52] demonstrated favorable effect directions on presenteeism outcomes in the intervention group compared with the control group. Among these, one study [52] targeted sedentary behavior, while the other [51] targeted both physical activity and sedentary behavior. Regarding the mHealth components, one [52] used mobile apps as part of the intervention, whereas the other [51] conducted a web-based mHealth intervention.

#### Impact of mHealth Interventions Primarily Targeting Physical Activity on Presenteeism Outcomes

No included studies examined the impact of mHealth interventions mainly targeting physical activity on presenteeism outcomes.

#### Impact of mHealth Interventions Primarily Targeting Sedentary Behavior on Presenteeism Outcomes

The cluster RCT [52], described in detail in the absenteeism section, also assessed presenteeism using the WLQ questionnaire. A favorable effect direction for overall work-related sickness presenteeism (higher scores indicate less sickness presenteeism) was observed (1/1, 100%), with group differences favoring the intervention group at 3 months (*β* coefficient=0.25, 95% CI 0.01-0.49; *P*=.04), 6 months (*β* coefficient=0.10, 95% CI −0.15 to 0.36; *P*=.42), and 12 months (*β* coefficient=0.25, 95% CI −0.08 to 0.58; *P*=.14) [52]. This study used mobile apps as mHealth technologies as part of the intervention.

#### Impact of mHealth Interventions Primarily Targeting Both Physical Activity and Sedentary Behavior on Presenteeism Outcomes

The cluster RCT [51], described in detail in the work productivity section, assessed presenteeism and found a favorable effect on presenteeism in the web-based intervention group compared with the active control group (1/1, 100%). This study assessed presenteeism using 3 domains for presenteeism in the WLQ, namely difficulty in meeting scheduling demands (time), performance of cognitive and interpersonal tasks (mental-interpersonal), and decreases in fulfilling the expected quantity, quality, and timeliness of completed work (output). Results showed a statistically significant 2 (group)×2 (program time points) interaction for the time (*P*_interaction_=.005), mental-interpersonal (*P*_interaction_=.019), and output scales for presenteeism (*P*_interaction_=.036). In both the intervention group and active comparison group, presenteeism was statistically significantly increased across the program, showing a universal increase in the difficulty of meeting scheduling demands (time scale), doing cognitive tasks and interacting with others (mental-interpersonal scale), and in meeting requirements for the quantity and quality of completed work (output scale). However, the intervention group consistently showed lower presenteeism impairment compared to the active comparison group across time points.

### Health-Related Outcomes

Ten included studies reported results related to physical activity [42-44,48-52,55,56] (Table S1 in Multimedia Appendix 3). Among these, 8 studies (80%) [42,43,49-52,55,56] demonstrated favorable effect directions on physical activity–related outcomes, whereas one study (10%) [48] showed no clear effect direction and another (10%) [44] showed an unfavorable effect direction. The majority of studies with favorable effect directions on physical activity–related outcomes (5/8, 62.5%) [42,49,50,55,56] used mHealth interventions combining mobile apps with wearable activity monitors or trackers. Of the remaining 3 studies, 1 [52] used mobile apps and 2 [43,51] used web-based components as mHealth tools for the intervention. Regarding sedentary behavior, 7 included studies reported relevant outcomes [43,48-52,56]. Of these, 6 studies (85.7%) [43,48,49,51,52,56] demonstrated favorable effect directions for sedentary behavior outcomes, whereas one study (14.3%) [50] showed no clear effect. Of these 6 studies [43,48,49,51,52,56], 1 [52] used mobile apps, 2 [43,51] used web-based components, and 3 [48,49,56] used combinations of mobile apps and wearable activity monitors or trackers as mHealth tools in the intervention. Of the 9 studies showing favorable effect directions on physical activity and/or sedentary behavior–related outcomes [42,43,48-52,55,56], all except one [51] implemented blended intervention strategies that included intervention components beyond mHealth technologies or tools.

### RoB Assessment

**Figure 2.**
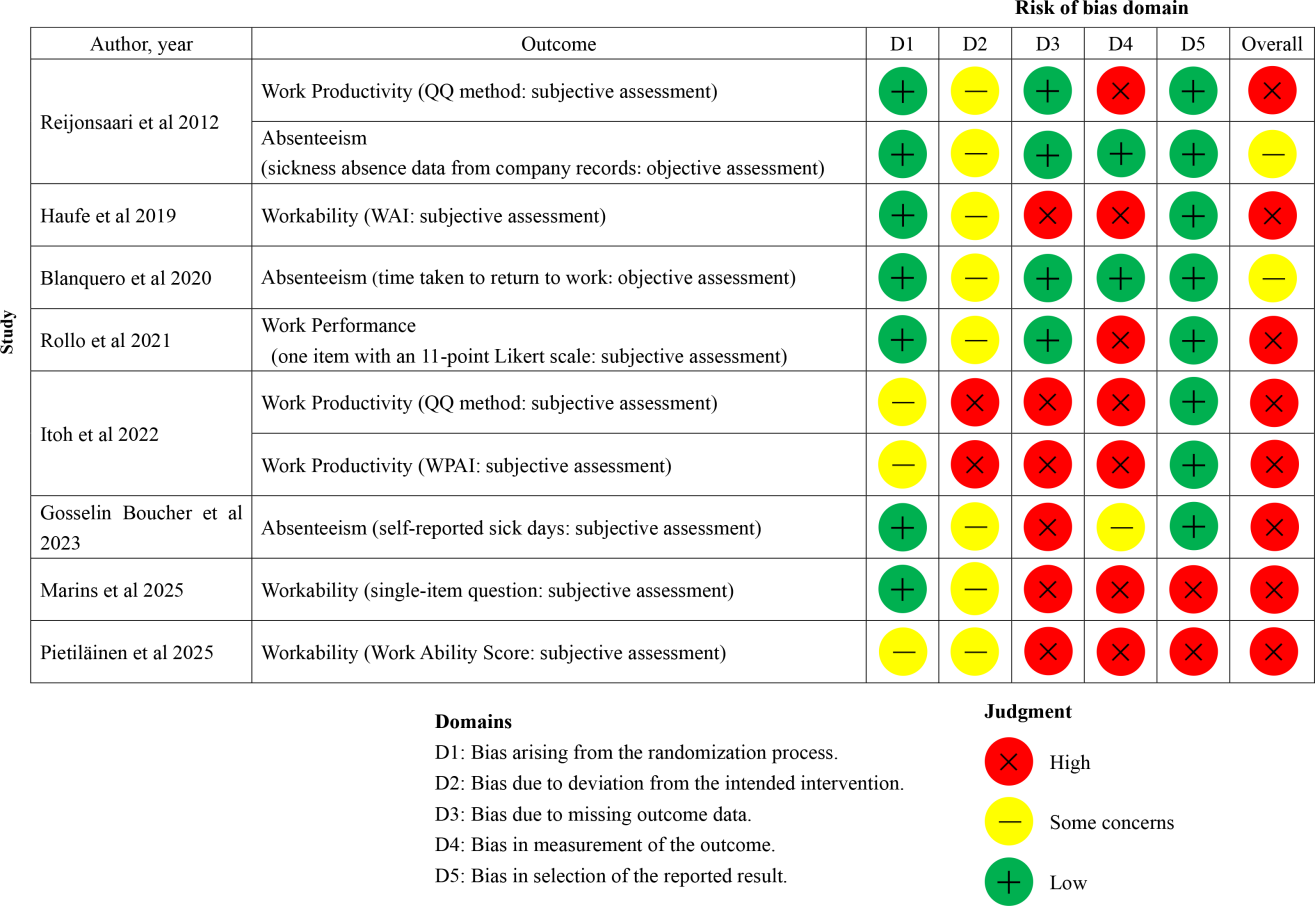
Risk of bias assessment for randomized controlled trials at the outcome level [41-48]. QQ method: quantity and quality method, WAI: Work Ability Index, WPAI: Work Productivity and Activity Impairment.

**Figure 3.**
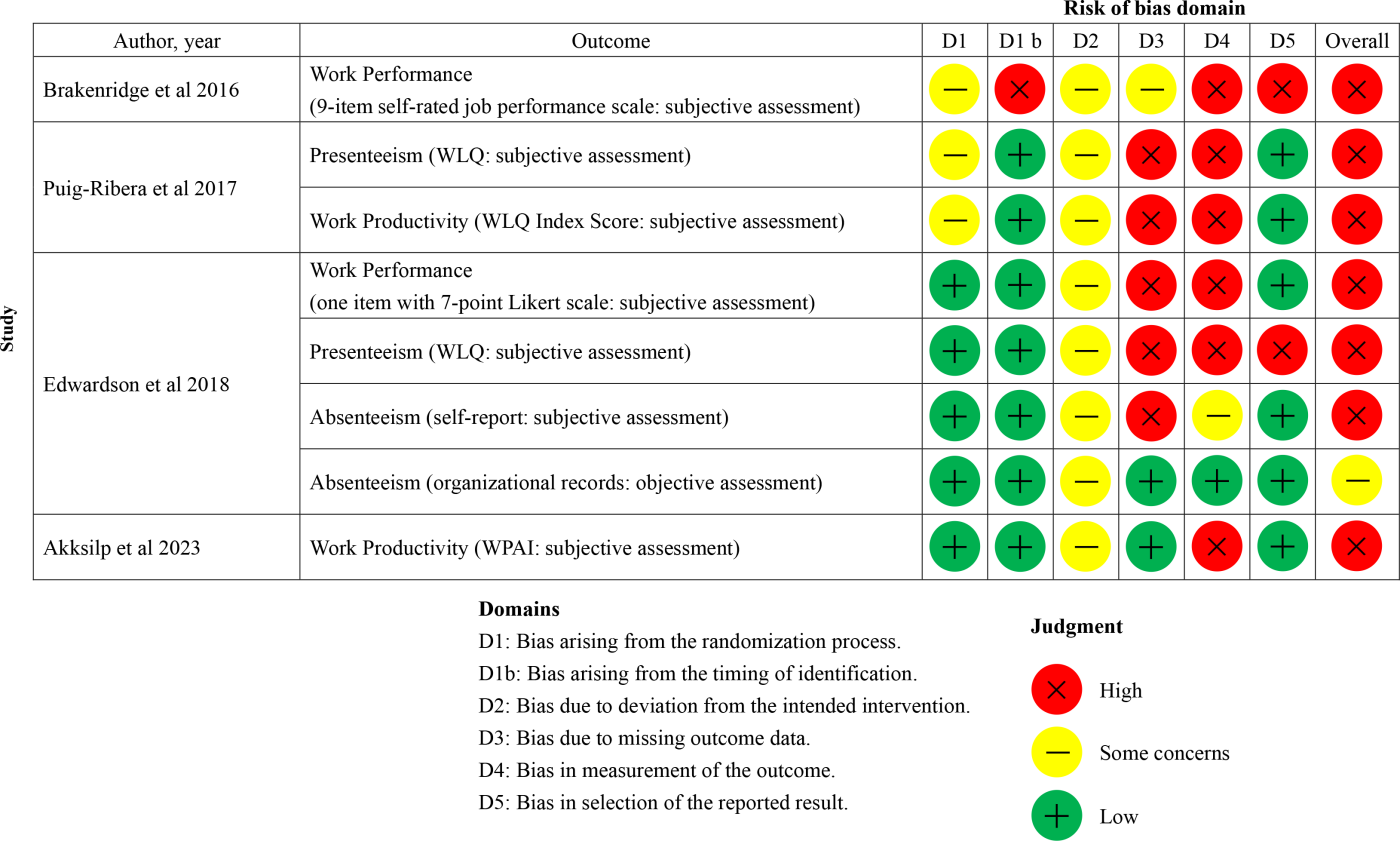
Risk of bias assessment for cluster randomized controlled trials at the outcome level [49-52]. WLQ: Work Limitations Questionnaire, WPAI: Work Productivity and Activity Impairment.

**Figure 4.**
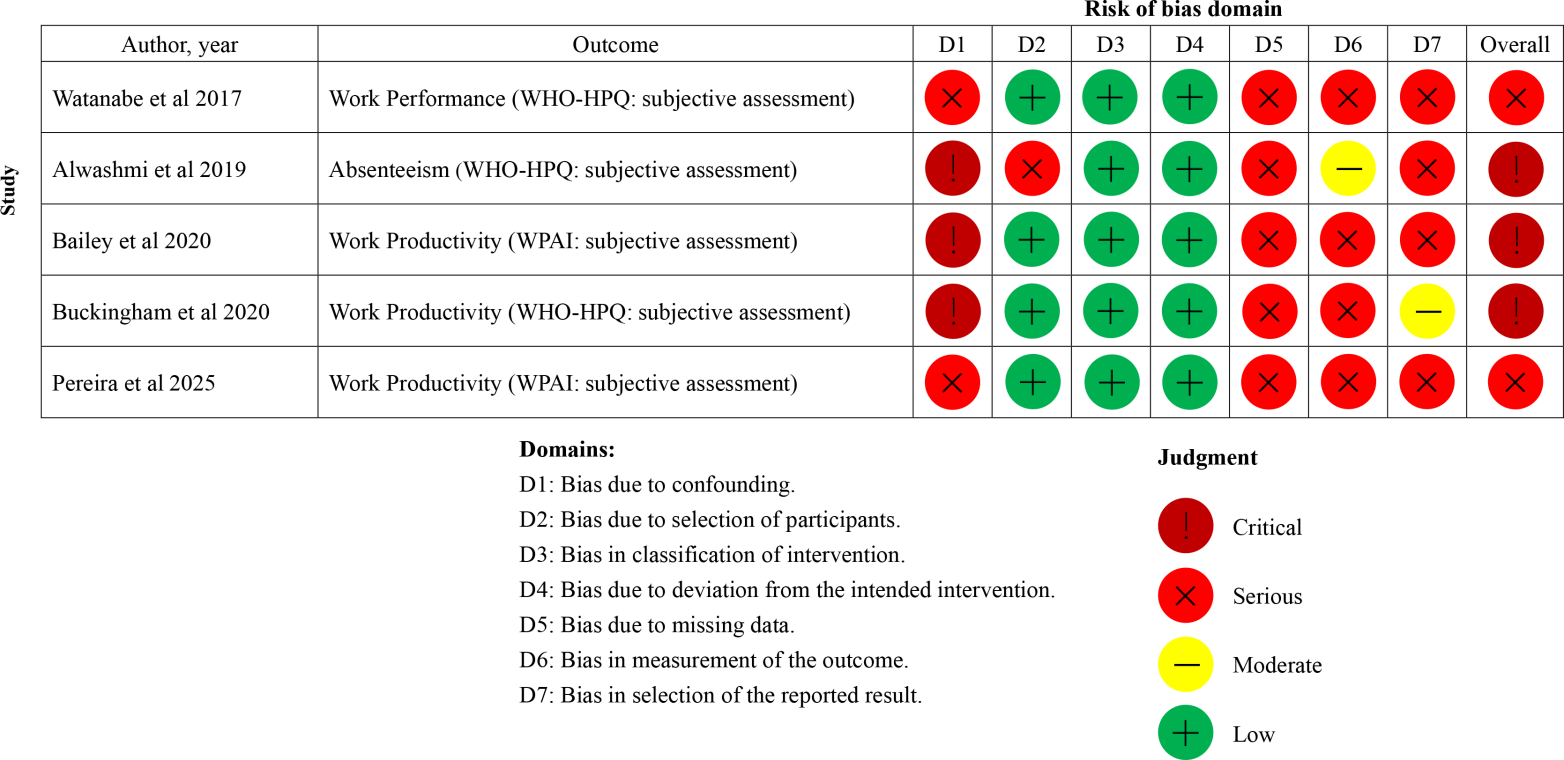
Risk of bias assessment for nonrandomized studies of interventions at the outcome level [53-57]. WHO-HPQ: World Health Organization Health and Work Performance Questionnaire; WPAI: Work Productivity and Activity Impairment.

Our RoB assessment for the included studies is shown in Figures 2-4. RoB was evaluated for each work-related outcome. In cases where a single study reported multiple work-related outcomes (eg, work absence evaluated using both organizational records and a self-reported questionnaire), the RoB for each outcome is presented individually. In the 12 RCT studies [41-52], 18 work-related outcomes were identified (work productivity [n=5], absenteeism [n=5], work performance [n=3], workability [n=3], and presenteeism [n=2]). Regarding the overall RoB in the included RCT studies (Figures2  3), 15 of 18 outcomes were rated as having a high RoB [41-45,47-52] and 3 as raising some concerns [44,46,52]. Of the 18 work-related outcomes, 14 were evaluated as either secondary [41-43,45,47,48,50-52] or tertiary outcomes [49], and only 4 were assessed as primary outcomes [41,44,46]. Consequently, the main domains of high risk of bias in many RCT studies were missing outcome data, outcome measurement, and selection of the reported result. In addition, given the nature of mHealth interventions, the included studies were necessarily conducted as open-label trials, raising concerns about the RoB due to deviations from the intended intervention. Domain 2 of the RoB 2, which assesses bias due to deviations from the intended intervention, was assessed as high risk or of some concerns in all RCT studies [41-52]. The overall risk of bias assessment for the 5 studies with an NRSI design [53-57] is summarized in Figure 4. In these 5 studies [53-57], a total of 5 work-related outcomes were identified (ie, work productivity [n=3], absenteeism [n=1], and work performance [n=1]). Regarding the overall risk of bias in these 5 studies [53-57], 3 outcomes were rated as at critical risk of bias [54-56] and 2 as at serious risk of bias [53,57]. The main domain of critical risk of bias was bias due to confounding.

### Adverse Events

Of the 17 included studies [41-57], adverse events were reported in 8 studies (47.1%; in one study [49], adverse event information was obtained from the corresponding author) [42,44,47,49,50,54,56,57], whereas no information on adverse events was provided in the remaining 9 studies (52.9%) [41,43,45,46,48,51-53,55]. The most frequent adverse events included skin reactions (including rashes, itching, and mild irritation) associated with the use of activity monitors and trackers, with the majority of adverse events being relatively minor. There were no reports of serious adverse events attributable to the intervention.

## Discussion

### Main Findings

This systematic review is, to our knowledge, the first to comprehensively examine the impact of mHealth interventions designed to encourage physical activity and decrease sedentary behavior on a wide range of work-related outcomes, including absenteeism, presenteeism, work productivity, work performance, and workability in working populations. A total of 17 studies (12 RCTs and 5 NRSIs) [41-57] met the eligibility criteria. Of these, 12 studies (70.6%) [41-43,45,46,48,49,51,52,54,55,57] reported favorable effect directions on at least one of the target work-related outcomes relative to control or comparison groups. However, the majority exhibited a high or critical risk of bias, raising concerns about the quality of the research. The considerable heterogeneity and overall low methodological quality of the included studies suggest that confidence in the reported effects of mHealth interventions aimed at promoting physical activity and reducing sedentary behavior on work-related outcomes remains limited at this stage. Considering these issues, it was not possible to reach definitive conclusions about the effectiveness of mHealth interventions on work-related outcomes.

### Summary of Findings on Work-Related Outcomes

Overall, 12 studies reported favorable effect directions for at least one targeted work-related outcome [41-43,45,46,48,49,51,52,54,55,57], 4 reported no clear effect direction [44,47,50,56], and 1 reported an unfavorable effect direction [53]. One study [53] observed that work performance decreased to a slightly greater extent among Pokémon GO players than among nonplayers, although the difference was not statistically significant. However, the majority of included studies indicated that mHealth intervention strategies targeting physical activity and/or sedentary behavior showed a favorable effect direction on work-related outcomes compared with control or comparison groups. Generally similar findings were observed across mHealth intervention types (ie, physical activity, sedentary behavior, or both). These findings suggest that health promotion efforts that incorporate mHealth components to encourage physical activity and/or decrease sedentary behavior are unlikely to have a substantial negative impact on work-related outcomes and have the potential to positively impact these outcomes. This finding is important as it provides useful evidence for stakeholders faced with decision-making about health promotion efforts incorporating mHealth components which encourage physical activity and decrease sedentary behavior among workers, in consideration of aspects of work-related outcomes.

### Presenteeism, Work Productivity, Work Performance, and Workability

In addition to the above issues, the use of various questionnaires to assess work-related outcomes such as presenteeism, work productivity, work performance, and workability has made it difficult to compare results across studies [60-62], and thereby hindered the ability to draw clear conclusions. Most of the included studies did not use the same questionnaires, and in some cases, identical questionnaires were used to assess different outcomes. For example, the WHO-HPQ was implemented to measure work productivity, presenteeism, and work performance [19]. Work productivity was classified as the efficiency and effectiveness of employees in performing their work, in consideration of the time (presenteeism) or resources (performance) required to perform a specific task, and the problems that required resolution in achieving their objectives [63]. Accordingly, the work productivity outcomes included both performance and presenteeism variables as a result of the two variables expressing a similar concept, albeit with the use of different tools [63,64]. In the context of workability, three included studies [42,47,48] assessed this outcome, but each used a different measurement tool. The use of different tools to measure these work-related outcomes makes it difficult to compare study results and draw definitive conclusions about the impact of mHealth intervention strategies.

### Absenteeism

Of the five included studies [44-46,52,55] that evaluated absenteeism outcomes, three studies targeting physical activity (two RCTs [45,46] and one retrospective cohort study [55]) demonstrated a favorable effect direction for absenteeism compared to the control or comparison groups. Absenteeism is defined as a period during which employees are absent from work due to illness or disability and is measured as the number of work hours or days missed [64]. Thus, this outcome can be objectively evaluated with materials such as workplace personnel records, making it generally less heterogeneous and more consistent in evaluation compared to other work-related outcomes. In fact, the risk of bias was less severe in those included studies that objectively evaluated absenteeism (eg, using organizational records) [44,46,52] compared to those that assessed it subjectively (eg, using self-reported measures) [45,52,55]. Among the three studies reporting a favorable direction of effect [45,46,55], only one [46] objectively assessed absenteeism, with the risk of bias assessed as being of some concerns. In contrast, the other two, which relied on subjective assessments, were judged to have a high [45] and a critical [55] risk of bias. In sum, mHealth interventions that promote physical activity may have the potential to decrease absenteeism; however, existing evidence on this issue is limited and raises concerns about bias risk. Therefore, further high-quality research is required before any definitive conclusions can be reached.

### Comparison With Previous Evidence

Since this systematic review is the first to explore the impact of mHealth interventions targeting physical activity promotion and sedentary behavior reduction with work-related outcomes, our results cannot be directly compared with those of previous systematic reviews. Nevertheless, the results are supported by evidence from prior systematic reviews on related topics, which were not restricted to mHealth-based intervention strategies. Consistent with this review, a prior systematic review investigating the effect of workplace nutrition and physical activity interventions on work-related outcomes suggested their potential effectiveness, and 14 studies showed statistically significant favorable changes in absenteeism (n=7), work performance (n=2), workability (n=3), work productivity (n=1), and both workability as well as work productivity (n=1) [19]. Likewise, another systematic review confirmed that a number of studies have demonstrated the effectiveness of worksite programs based on physical activity in improving work-related outcomes, such as workability (n=5), absenteeism (n=3), and work productivity (n=5) [20]. The consistency between our findings and those of previous studies supports the reliability of the present results and strengthens the notion that health promotion efforts incorporating mHealth technologies to promote physical activity and reduce sedentary behavior may positively impact work-related outcomes. Notably, earlier systematic reviews [19,20] primarily focused on traditional workplace-based interventions and did not consider mHealth technologies as key components of intervention. Implementation barriers commonly reported for workplace health promotion programs include financial constraints, limited personnel or expertise, and insufficient physical space [21]. mHealth technologies may help address these challenges by reducing reliance on on-site resources and enabling scalable, flexible, and context-independent intervention delivery beyond conventional workplace boundaries. The importance of scalability for broad implementation has been emphasized in health promotion research [14]. By synthesizing recent evidence, this review contributes to the existing literature by expanding the scope of intervention settings (eg, not only workplace settings but also home and outdoor contexts) and by emphasizing studies in which mHealth technologies constituted key components of the interventions, thereby enhancing their scalability and potential for broader implementation.

### mHealth Strategies and Components

The included studies covered a wide range of mHealth strategies. The most frequently reported mHealth technology was mobile apps, used either alone or in combination with wearable activity monitors or trackers (14/17, 82.4%) [41,42,45-50,52-57]. Even when categorized under the same type of mHealth technology, the content and design of each mHealth intervention varied substantially. In addition, many intervention strategies included not only mHealth components but also other components, such as human support (eg, personal counseling; 13/17, 76.5%) [41-44,46,48-50,52,54-57], which further contributed to heterogeneity across studies. This makes it difficult to attribute the effects of the interventions solely to the mHealth technologies. It has been suggested that adherence to interventions is low when mHealth is used as a standalone methodology [30]. In contrast, personal communication with and integration of health care professionals has been reported to positively influence adherence to mHealth interventions focusing on physical activity [30]. In fact, in one included study using an mHealth-only intervention [45], app use declined over the 12-week intervention, with only 33 participants (33/142, 23%) continuing to use the apps regularly by the end of the trial. In the included studies, no information on adherence—defined as the extent to which users followed the program as designed—was reported for mHealth-only interventions, except for one study [45]. Consequently, adherence could not be compared between mHealth-only and blended interventions, and the details remain unclear. Nevertheless, the potentially low adherence to mHealth-only interventions may partly explain why many studies incorporated additional components rather than relying solely on mHealth technologies, which is consistent with previous literature [30]. Likewise, the NICE committee recommended considering digital and mHealth interventions as options to increase physical activity alongside existing services, while noting that their effectiveness is variable [32]. Taken together, these results indicate that mHealth interventions should be viewed as a complement to existing services, such as human support, rather than a replacement for them, both in future research and in the deployment of mHealth technologies in real-world settings.

### Strengths

This systematic review has several strengths. First, it used a comprehensive search strategy across major electronic databases, facilitating an evidence-based approach to the literature search. Second, it included literature on studies using not only RCT but also NRSI designs. Incorporating non-RCT study designs provides broader evidence to guide the implementation of interventions in real-world settings, particularly in situations where conducting an RCT is difficult or inappropriate. Third, each study included in this review was selected and assessed in a comprehensive manner, and its data were extracted and assessed for quality by 2 researchers acting independently in order to remove potential biases in the review process. Additionally, we used 2 methodological quality assessment tools recommended by the Cochrane Collaboration to assess the risk of bias in the included RCTs and NRSIs.

### Limitations

We also note a number of limitations of this review. First, a major limitation of this review is the substantial heterogeneity across study populations, interventions, and outcomes, which precluded meta-analysis and prevented a reliable assessment of the overall certainty of the evidence using the GRADE approach. Instead, this review relied on a narrative synthesis based on vote counting of effect directions across studies. While this approach can indicate effect direction, it cannot estimate the overall effect size across studies. This limitation may reduce the strength of the conclusions and impede the ability to identify which interventions are most effective for work-related outcomes, and the conditions under which they are most beneficial. Future studies using standardized outcome measures and comparable interventions would enable quantitative synthesis of the evidence through meta-analysis, and contribute to addressing this limitation. Second, the review was limited to papers written in English and Japanese, introducing a degree of language bias. However, we confirmed that there were no studies within the screened literature other than those in Japanese and English that met the inclusion criteria for this systematic review. Additionally, some studies that were potentially both useful and relevant may have been excluded because they were not full-text original articles, such as conference proceedings, and unpublished manuscripts. To minimize this possibility, we reviewed the content of non–full-text original articles identified during screening, such as conference proceedings, trial registry records, and protocol papers, to confirm whether any corresponding full-text original studies relevant to this review had been published. Moreover, only 2 of the included studies [41,47] used the CONSORT-EHEALTH checklist [65] or the reporting guidelines for mHealth interventions [66], which may hinder the successful implementation of effective interventions in real-world settings. Finally, many of the included studies in this review investigated relatively short-term effects, with the duration of the interventions ranging from 6 weeks to 12 months, and the long-term impact on work-related outcomes therefore remains unclear.

### Implications for Future Research

What future research is necessary to advance this field beyond the results of this systematic review? First, a key priority is to promote further high-quality research that ensures a low possibility of bias risk. Many of the included studies were evaluated as having a high or critical risk of bias, including not only NRSIs [54-56] but also RCTs [41-45,47-52]. Among the included studies, the majority of work-related outcomes were evaluated as either secondary [41-43,45,47,48,50-57] or tertiary outcomes [49], and only 4 outcomes were evaluated as primary outcomes [41,44,46]. This may have partly contributed to a high risk of bias across the domains of missing outcome data, outcome measurement, and selection of the reported results. A high proportion of missing data may reduce the reliability and generalizability of the findings. In addition, selective reporting of outcomes as well as outcome measurement based on self-reports could lead to an overestimation of intervention effects. Future research that assesses work-related outcomes as a primary outcome may help address these bias-related concerns. From other perspectives, efforts to increase adherence will likely help reduce the risk of bias due to deviation from the intended intervention. The degree to which the study intervention was implemented as intended is one of the most challenging issues in the field of mHealth interventions [31]. Addressing low adherence rates is essential, as low adherence can reduce statistical power and introduce bias, particularly by making a potentially efficacious intervention appear less effective, thereby leading to misleading recommendations in mHealth interventions [31]. While low adherence rates in mHealth interventions have recently been widely recognized, strategies that may address these difficulties are emerging [30,31]. Examples that have been shown to positively influence adherence to mHealth interventions focusing on physical activity include customizable push notifications, gamification, social features (eg, competition, social comparison, and challenges), app personalization and customization, personal communication with, and integration of, health care professionals [30]. Future studies incorporating this emerging evidence on adherence to mHealth tools may help establish effective intervention strategies that encourage physical activity and decrease sedentary behavior, thereby contributing to improvements in work-related outcomes. Finally, in addition to the challenges mentioned above, a major limitation of the current evidence is the substantial heterogeneity among the included studies, which prevents quantitative synthesis through meta-analysis. Addressing such heterogeneity might require innovative approaches such as mega-trials, where various interventions are tested simultaneously in identical populations with uniform measurement periods and consistent outcome assessments [67]. Alternatively, promoting future research that adheres to frameworks such as the CONSORT-EHEALTH checklist [65] and the reporting guidelines for mHealth interventions [66] may contribute to standardization across studies, and thereby help overcome heterogeneity among them. As an additional benefit, compliance with these checklists and guidelines could enhance research quality and, furthermore, ensure the reproducibility of the intervention, which is itself essential for the successful implementation of intervention strategies in real-world settings. It will also be valuable to observe which apps developed in research settings successfully transition to the market, while evaluating their impact and quality using tools such as the Mobile App Rating Scale (MARS) [68]. In one included study [56], a clear association was observed between engagement (defined by usage) and perceived usability and usefulness, suggesting that incorporating user feedback is essential for developing effective interventions in real-world settings.

### Conclusions

In conclusion, this systematic review suggests that health promotion initiatives incorporating mHealth components to promote physical activity and reduce sedentary behavior may have a positive impact on work-related outcomes. However, the current evidence is limited and generally of low quality, due to a lack of high-quality studies and substantial heterogeneity in study populations, intervention content, outcome measures, and study designs. To draw more reliable conclusions, future research should include large-scale, high-quality studies with long-term follow-up and objective or validated outcome assessments. This review is the first to comprehensively synthesize evidence on work-related outcomes, extending prior reviews that primarily focused on traditional workplace-based interventions without mHealth components. The findings of this review may provide guidance for future studies, including the potential value of megastudy designs that evaluate multiple interventions within the same population over the same period on the same outcomes. In real-world settings, these findings suggest that mHealth technologies may serve as a complementary strategy for supporting occupational health and productivity.

## Supplementary material

10.2196/80540 Multimedia Appendix 1 Search terms used in electronic database searches.

10.2196/80540 Multimedia Appendix 2 List of excluded studies along with reasons for exclusion.

10.2196/80540 Multimedia Appendix 3 Extracted data and supplementary tables S1 and S2.

10.2196/80540 Multimedia Appendix 4 Synthesis Without Meta-Analysis (SWiM) in systematic reviews: reporting guidelines checklist.

10.2196/80540 Checklist 1 PRISMA 2020 checklist.

10.2196/80540 Checklist 2 PRISMA-S checklist for reporting literature searches.

## Abbreviations

CENTRAL: Cochrane Central Register of Controlled Trials
GRADE: Grading of Recommendations Assessment, Development and Evaluation
ICTRP: International Clinical Trials Registry Platform
MARS: Mobile App Rating Scale
MeSH: Medical Subject Headings
mHealth: mobile health
NICE: National Institute for Health and Care Excellence
NRSI: nonrandomized study of interventions
PFMT: pelvic floor muscle training
PICO: Population, Intervention, Comparison, and Outcome
PRISMA: Preferred Reporting Items for Systematic reviews and Meta-Analyses
PRISMA-S: Preferred Reporting Items for Systematic Reviews and Meta-Analyses literature search extension
RCT: randomized controlled trial
RoB: risk of bias
ROBINS-I: Risk Of Bias In Nonrandomized Studies of Interventions
SWiM: Synthesis Without Meta-Analysis
WHO: World Health Organization
WHO-HPQ: World Health Organization Health and Work Performance Questionnaire
WLQ: Work Limitation Questionnaire
WPAI: Work Productivity and Activity Impairment
