# Supplementary material for: Effectiveness of mHealth Interventions Aimed at Promoting Physical Activity and Reducing Sedentary Behavior on Work-Related Outcomes Among Workers: Systematic Review
Source: J Med Internet Res. 2026 May 7;28:e80540. doi: 10.2196/80540 (PMC13152228; doi:10.2196/80540)
Supplement: Multimedia Appendix 1 [file jmir-v28-e80540-s001.docx]

Multimedia Appendix 1. Search terms used in electronic database searches.

Database: MEDLINE (via PubMed)

*Search Date: September 23, 2023 (original); September 12, 2025 (updated).*

| ***Id*** | **Criteria** |
| --- | --- |
| #1 | "Computers, Handheld"[MeSH Terms] OR ("computers"[All Fields] AND "handheld"[All Fields]) OR "handheld computers"[All Fields] OR ("computers"[All Fields] AND "handheld"[All Fields]) OR "computers, handheld"[All Fields] |
| #2 | "Wireless Technology"[MeSH Terms] OR ("wireless"[All Fields] AND "technology"[All Fields]) OR "wireless technology"[All Fields] |
| #3 | "Wearable Electronic Devices"[MeSH Terms] OR ("wearable"[All Fields] AND "electronic"[All Fields] AND "devices"[All Fields]) OR "wearable electronic devices"[All Fields] |
| #4 | mobile[tiab] OR smart*[tiab] OR tablet?[tiab] OR smartphone?[tiab] OR smartwatch*[tiab] OR tablet computer?[tiab] OR electronic tablet?[tiab] OR electronic device?[tiab] OR wireless communication?[tiab] OR ipad?[tiab] OR i-pad?[tiab] OR iphone?[tiab] OR i-phone?[tiab] OR android[tiab] OR wearable?[tiab] OR Internet of things[tiab] OR IOT[tiab] OR cell-phone[tiab] OR "Cell Phone"[MeSH Terms] |
| #5 | OR/#1-#4 |
| #6 | App?[tiab] OR application?[tiab] OR software[tiab] OR "Mobile Applications"[MeSH Terms] |
| #7 | (("digital"[All Fields] OR "digitalisation"[All Fields] OR "digitalised"[All Fields] OR "digitalization"[All Fields] OR "digitalize"[All Fields] OR "digitalized"[All Fields] OR "digitalizer"[All Fields] OR "digitalizing"[All Fields] OR "digitally"[All Fields] OR "digitals"[All Fields] OR "digitization"[All Fields] OR "digitizations"[All Fields] OR "digitize"[All Fields] OR "digitized"[All Fields] OR "digitizer"[All Fields] OR "digitizers"[All Fields] OR "digitizes"[All Fields] OR "digitizing"[All Fields]) AND "biomark"[All Fields]) OR ("Track and Field"[MeSH Terms] OR ("track"[All Fields] AND "field"[All Fields]) OR "track and field"[All Fields] OR "track"[All Fields] OR "tracks"[All Fields] OR "tracked"[All Fields] OR "tracking"[All Fields] OR "trackings"[All Fields]) OR ("monitor"[All Fields] OR "monitors"[All Fields] OR "monitorable"[All Fields] OR "monitored"[All Fields] OR "monitoring"[All Fields] OR "monitorings"[All Fields] OR "monitorings"[All Fields] OR "monitorization"[All Fields] OR "monitorize"[All Fields] OR "monitorized"[All Fields] OR "monitors"[All Fields]) OR ("sensor"[All Fields] OR "sensors"[All Fields] OR "sensoric"[All Fields] OR "sensorics"[All Fields] OR "sensoring"[All Fields] OR "sensorization"[All Fields] OR "sensorized"[All Fields] OR "sensors"[All Fields]) OR ("nanosensor"[All Fields] OR "nanosensors"[All Fields]) OR "ResearchKit"[All Fields] OR "ResearchStack"[All Fields] OR "HealthKit"[All Fields] OR "CareKit"[All Fields] OR "Google Fit"[All Fields] OR "Apple Health"[All Fields] |
| #8 | "Monitoring, Physiologic"[MeSH Terms] OR ("monitoring"[All Fields] AND "physiologic"[All Fields]) OR "physiologic monitoring"[All Fields] OR ("monitoring"[All Fields] AND "physiologic"[All Fields]) OR "monitoring, physiologic"[All Fields] |
| #9 | "Self Care"[MeSH Terms] OR ("self"[All Fields] AND "care"[All Fields]) OR "self care"[All Fields] |
| #10 | "Self-Management"[MeSH Terms] OR "self-management"[All Fields] OR ("self"[All Fields] AND "management"[All Fields]) OR "self management"[All Fields] |
| #11 | "mhealths"[All Fields] OR "mhealth"[All Fields] OR "m-health"[All Fields] OR (("electronical"[All Fields] OR "electronically"[All Fields] OR "Electronics"[MeSH Terms] OR "electronics"[All Fields] OR "electronic"[All Fields]) AND ("Health"[MeSH Terms] OR "health"[All Fields] OR "healths"[All Fields] OR "healthful"[All Fields] OR "healthfulness"[All Fields] OR "healths"[All Fields])) OR ("ehealth"[All Fields]) OR "e-health"[All Fields] OR "digital health"[tiab] OR ("digital"[tiab] AND "health"[tiab]) |
| #12 | "Health Records, Personal"[MeSH Terms] OR ("health"[All Fields] AND "records"[All Fields] AND "personal"[All Fields]) OR "personal health records"[All Fields] OR ("personal"[All Fields] AND "health"[All Fields] AND "record"[All Fields]) OR "personal health record"[All Fields] |
| #13 | "Medical Informatics"[MeSH Terms] OR ("medical"[tiab] AND "informatics"[tiab]) OR "medical informatics"[tiab] |
| #14 | OR/#6-#13 |
| #15 | "Exercise"[MeSH Terms] OR "exercise"[tiab] OR "exercises"[tiab] OR "Exercise Therapy"[MeSH Terms] OR ("exercise"[tiab] AND "therapy"[tiab]) OR "exercise therapy"[tiab] |
| #16 | "Exercise Therapy"[MeSH Terms] OR ("exercise"[All Fields] AND "therapy"[All Fields]) OR "exercise therapy"[All Fields] |
| #17 | "Physical Fitness"[MeSH Terms] OR ("physical"[tiab] AND "fitness"[tiab]) OR "physical fitness"[tiab] OR "physical activit*"[tiab] |
| #18 | "Exercise Movement Techniques"[MeSH Terms] OR ("exercise"[tiab] AND "movement"[tiab] AND "techniques"[tiab]) OR "exercise movement techniques"[tiab] |
| #19 | "Leisure Activities"[MeSH Terms] OR ("leisure"[tiab] AND "activities"[tiab]) OR "leisure activities"[tiab] OR ("leisure"[All Fields] AND "activity"[tiab]) OR "leisure activity"[tiab] |
| #20 | "Fitness Trackers"[MeSH Terms] OR ("fitness"[All Fields] AND "trackers"[All Fields]) OR "fitness trackers"[All Fields] OR ("fitness"[All Fields] AND "tracker"[All Fields]) OR "fitness tracker"[All Fields] |
| #21 | "Physical Education and Training"[MeSH Terms] OR ("physical"[All Fields] AND "education"[All Fields] AND "training"[All Fields]) OR "physical education and training"[All Fields] |
| #22 | "Sedentary Behavior"[MeSH Terms] OR ("sedentary"[All Fields] AND "behavior"[All Fields]) OR "sedentary behavior"[All Fields] OR ("sedentary"[All Fields] AND "behaviour"[All Fields]) OR "sedentary behaviour"[All Fields] |
| #23 | OR/#15-#22 |
| #24 | #5 AND #14 AND #23 |
| #25 | "Occupational Groups"[MeSH Terms] OR "occupational"[All Fields] OR "occupational groups"[All Fields] OR "worker*"[All Fields] OR "worksite*"[All Fields] OR "workplace*"[All Fields] OR "employ*"[All Fields] |
| #26 | #24 AND #25 |
| #27 | "absentee"[All Fields] OR "Absenteeism"[MeSH Terms] OR "absenteeism"[All Fields] OR "absentees"[All Fields] |
| #28 | "Work Performance"[MeSH Terms] OR ("work"[All Fields] AND "performance"[All Fields]) OR "work performance"[All Fields] |
| #29 | "Presenteeism"[MeSH Terms] OR "presenteeism"[All Fields] |
| #30 | "Sick Leave"[MeSH Terms] OR ("sick"[All Fields] AND "leave"[All Fields]) OR "sick leave$"[All Fields] |
| #31 | "Economics"[MeSH Terms] OR "economics"[All Fields] OR "production"[All Fields] OR "productions"[All Fields] OR "Efficiency"[MeSH Terms] OR "efficiency"[All Fields] OR "productivity"[All Fields] OR "product"[All Fields] OR "product's"[All Fields] OR "productive"[All Fields] OR "productively"[All Fields] OR "productivities"[All Fields] OR "products"[All Fields] |
| #32 | "workability"[All Fields] OR "work ability" |
| #33 | "Quality of Life"[MeSH Terms] OR ("quality"[All Fields] AND "life"[All Fields]) OR "quality of life"[All Fields] |
| #34 | "Occupational Health"[MeSH Terms] OR ("occupational"[All Fields] AND "health"[All Fields]) OR "occupational health"[All Fields] |
| #35 | "efficiences"[All Fields] OR "Efficiency"[MeSH Terms] OR "efficiency"[All Fields] OR "efficiencies"[All Fields] OR "efficient"[All Fields] OR "efficiently"[All Fields] OR "efficients"[All Fields] |
| #36 | OR/#27-#35 |
| #37 | #26 AND #36 |

MeSH, Medical Subject Headings.

Database: Web of Science Core Collection (via Web of Science)

*Search Date: September 23, 2023 (original); September 12, 2025 (updated).*

| ***Id*** | **Criteria** |
| --- | --- |
| #1 | TS=(handheld computer$) |
| #2 | TS=(wireless technolog*) |
| #3 | TS=(wearable electronic device$) |
| #4 | TS=(mobile OR tablet$ OR smartphone$ OR smartwatch* OR electronic device$ OR wireless communication$ OR ipad$ OR i-pad$ OR iphone$ OR i-phone$ OR android OR wearable OR Internet of things OR IOT OR cell-phone) |
| #5 | #1 OR #2 OR #3 OR #4 |
| #6 | TS= (App$ OR application$ OR software) |
| #7 | TS=(digital biomark* OR track* OR monitor* OR sensor* OR nanosensor* OR ResearchKit OR ResearchStack OR HealthKit OR CareKit OR "Google Fit" OR "Apple Health") |
| #8 | TS=(monitoring, physiologic) |
| #9 | TS=(self care) |
| #10 | TS=(self management) |
| #11 | TS=(mhealth OR m-health OR electronic health OR ehealth OR e-health OR digital health) |
| #12 | TS=(personal health record) |
| #13 | TS=(medical informatics) |
| #14 | #6 OR #7 OR #8 OR #9 OR #10 OR #11 OR #12 OR #13 |
| #15 | TS=(exercise) |
| #16 | TS=(exercise therapy) |
| #17 | TS=(physical activit* OR fitness) |
| #18 | TS=(pilates OR yoga OR dance) |
| #19 | TS=(leisure activit*) |
| #20 | TS=(fitness tracker*) |
| #21 | TS=(physical education? OR training) |
| #22 | TS=(sedentary behavio$r OR sedentary OR sit*) |
| #23 | #15 OR #16 OR #17 OR #18 OR #19 OR #20 OR #21 OR #22 |
| #24 | #5 AND #14 AND #23 |
| #25 | TS=(occupational OR worker* OR worksite* OR workplace* OR employ*) |
| #26 | #24 AND #25 |
| #27 | TS=(absenteeism) |
| #28 | TS=(work performance) |
| #29 | TS=(presenteeism) |
| #30 | TS=(sick leave$ OR "paid sick" OR "sick pay") |
| #31 | TS=(productivity) |
| #32 | TS=(workability OR work ability) |
| #33 | TS=(quality of Life) |
| #34 | TS=(occupational Health) |
| #35 | TS=(efficiency) |
| #36 | #27 OR #28 OR #29 OR #30 OR #31 OR #32 OR #33 OR #34 OR #35 |
| #37 | #26 AND #36 |

Database: Cochrane Library (including Cochrane Central Register of Controlled Trials [CENTRAL] and Cochrane Database of Systematic Reviews)

*Search Date: September 23, 2023 (original); September 12, 2025 (updated).*

| ***Id*** | **Criteria** |
| --- | --- |
| #1 | MeSH descriptor: [Computers, Handheld] explode all trees |
| #2 | (computers handheld):ti,ab,kw (Word variations have been searched) |
| #3 | MeSH descriptor: [Wireless Technology] explode all trees |
| #4 | (wireless technology):ti,ab,kw (Word variations have been searched) |
| #5 | MeSH descriptor: [Wearable Electronic Devices] explode all trees |
| #6 | (wearable electronic devices):ti,ab,kw (Word variations have been searched) |
| #7 | MeSH descriptor: [Smartphone] explode all trees |
| #8 | MeSH descriptor: [Cell Phone] explode all trees |
| #9 | (mobile OR smart* OR tablet? OR smartphone? OR smartwatch* OR tablet computer? OR electronic tablet? OR electronic device? OR wireless communication? OR ipad? OR i-pad? OR iphone? OR i-phone? OR android OR wearable? OR "Internet of things" OR IOT or cell-phone):ti,ab,kw |
| #10 | {OR #1-#9} |
| #11 | MeSH descriptor: [Mobile Applications] explode all trees |
| #12 | MeSH descriptor: [Software] explode all trees |
| #13 | (App? OR application? OR software):ti,ab,kw |
| #14 | (digital biomark* OR track* OR monitor* OR sensor* OR nanosensor* OR ResearchKit OR ResearchStack OR HealthKit OR CareKit OR "Google Fit" OR "Apple Health"):ti,ab,kw |
| #15 | MeSH descriptor: [Monitoring, Physiologic] explode all trees |
| #16 | (monitoring physiologic):ti,ab,kw (Word variations have been searched) |
| #17 | MeSH descriptor: [Self Care] explode all trees |
| #18 | (self care):ti,ab,kw (Word variations have been searched) |
| #19 | MeSH descriptor: [Self-Management] explode all trees |
| #20 | (self management):ti,ab,kw (Word variations have been searched) |
| #21 | (mhealth OR m-health OR electronic health OR ehealth OR e-health OR digital health):ti,ab,kw (Word variations have been searched) |
| #22 | MeSH descriptor: [Health Records, Personal] explode all trees |
| #23 | (personal health record):ti,ab,kw (Word variations have been searched) |
| #24 | MeSH descriptor: [Medical Informatics] explode all trees |
| #25 | (medical informatics):ti,ab,kw (Word variations have been searched) |
| #26 | {OR #11-#25} |
| #27 | MeSH descriptor: [Exercise] explode all trees |
| #28 | exercise:ti,ab,kw (Word variations have been searched) |
| #29 | MeSH descriptor: [Exercise Therapy] explode all trees |
| #30 | (exercise therapy):ti,ab,kw (Word variations have been searched) |
| #31 | MeSH descriptor: [Physical Fitness] explode all trees |
| #32 | ((physical fitness) OR (physical activity)):ti,ab,kw (Word variations have been searched) |
| #33 | MeSH descriptor: [Exercise Movement Techniques] explode all trees |
| #34 | (exercise movement technique):ti,ab,kw (Word variations have been searched) |
| #35 | MeSH descriptor: [Leisure Activities] explode all trees |
| #36 | (leisure activity):ti,ab,kw (Word variations have been searched) |
| #37 | MeSH descriptor: [Fitness Trackers] explode all trees |
| #38 | (fitness tracker):ti,ab,kw (Word variations have been searched) |
| #39 | MeSH descriptor: [Physical Education and Training] explode all trees |
| #40 | (physical education OR training):ti,ab,kw (Word variations have been searched) |
| #41 | MeSH descriptor: [Sedentary Behavior] explode all trees |
| #42 | ("sedentary behaviour" OR "sedentary behavior" OR sedentary OR sit):ti,ab,kw (Word variations have been searched) |
| #43 | {OR #27-#42} |
| #44 | #10 AND #26 AND #43 |
| #45 | MeSH descriptor: [Occupational Groups] explode all trees |
| #46 | MeSH descriptor: [Workplace] explode all trees |
| #47 | (worker OR worksite OR workplace OR employ):ti,ab,kw (Word variations have been searched) |
| #48 | {OR #45-#47} |
| #49 | #44 AND #48 |
| #50 | MeSH descriptor: [Absenteeism] explode all trees |
| #51 | absenteeism: ti,ab,kw (Word variations have been searched) |
| #52 | MeSH descriptor: [Work Performance] explode all trees |
| #53 | (work performance): ti,ab,kw (Word variations have been searched) |
| #54 | MeSH descriptor: [Presenteeism] explode all trees |
| #55 | presenteeism: ti,ab,kw (Word variations have been searched) |
| #56 | MeSH descriptor: [Sick Leave] explode all trees |
| #57 | (sick leave OR "paid sick" OR "sick pay"): ti,ab,kw (Word variations have been searched) |
| #58 | MeSH descriptor: [Economics] explode all trees |
| #59 | (economics OR production OR product): ti,ab,kw (Word variations have been searched) |
| #60 | (workability OR "work ability"): ti,ab,kw (Word variations have been searched) |
| #61 | MeSH descriptor: [Quality of Life] explode all trees |
| #62 | ("quality of life" OR QOL): ti,ab,kw (Word variations have been searched) |
| #63 | MeSH descriptor: [Occupational Health] explode all trees |
| #64 | (occupational health): ti,ab,kw (Word variations have been searched) |
| #65 | MeSH descriptor: [Efficiency] explode all trees |
| #66 | efficiency: ti,ab,kw (Word variations have been searched) |
| #67 | {OR #50-#66} |
| #68 | #49 AND #67 |

Database: Ichushi-Web (Japan Medical Abstracts Society)

*Search Date: September 23, 2023 (original); September 12, 2025 (updated).*

| ***Id*** | **Criteria** |
| --- | --- |
| #1 | 携帯情報端末/TH or “Handheld Computer”/AL or ハンドヘルド/AL |
| #2 | 無線技術/TH or 無線技術/AL or ワイヤレステクノロジー/AL or “Wireless Technology”/AL |
| #3 | ウェアラブル電子機器/TH or “Wearable Electronic Device”/AL or (ウェアラブル/AL and 電子機器/AL) |
| #4 | (mobile/TA or Rモバイル/TA) or (smart/TA orスマート/TA) or (tablet/TA orタブレット/TA) or (smartphone/TA or スマートフォン/TA or スマートフォン/TH) or (smartwatch/ TA or スマートウォッチ/TA) or (電子機器/TA or “Electronic Device”/TA) or (無線/TA or ワイヤレス/TA or “Wireless communication”/TA) or (ipad/TA or i-pad/TA) or (iphone/TA or i-phone/TA) or (android/TA orアンドロイド/TA) or (Wearable/TA or ウェアラブル/TA) or ("IoT (モノのインターネット)"/TH or “Internet of Things”/TA or IOT/TA) or (cell-phone/TA OR 携帯電話/TA or 携帯電話/TH) |
| #5 | #1 or #2 or #3 or #4 |
| #6 | (App/AL or アプリ/AL) or (application/AL or アプリケーション/AL) or (software/AL or ソフト/AL or ソフトウェア/TH) or モバイルアプリケーション/TH |
| #7 | (デジタル/AL and バイオマーカー/AL) or (“digital biomark”/AL) or (track/AL or トラッカー/AL) or (monitor/AL or モニタ/AL or データディスプレイ/TH) or (センサー/TH or sensor/AL) or (nanosensor/AL or ナノセンサー/AL) or (ResearchKiT/AL or リサーチキット) or ResearchStack/AL or HealthKiT/AL or CareKiT/AL or “Google Fit”/AL or “Apple Health”/AL |
| #8 | 生体機能モニタリング/TH or “Physiologic Monitoring”/AL |
| #9 | “self care”/AL or セルフケア/AL or 自己管理/TH or 自己管理/AL |
| #10 | 慢性疾患セルフマネジメント/TH or “self management”/AL or セルフマネジメント/AL |
| #11 | (mhealth/AL or m-health/AL) or (“electronic health”/AL or ehealth/AL or e-health/AL) or (“digital health”/AL or デジタルヘルス/AL) or モバイルヘルス/AL |
| #12 | パーソナルヘルスレコード/TH or “Personal Health Record”/AL |
| #13 | 医療情報学/TH or “medical informatics”/AL |
| #14 | #6 or #7 or #8 or #9 or #10 or #11 or #12 or #13 |
| #15 | 身体運動/TH or exercise/AL or エクササイズ/AL or 運動/TA |
| #16 | 運動療法/TH |
| #17 | 体力/TH or 体力/AL or “physical fitness”/AL or “physical activity”/AL or “physical activities”/AL or 身体的活動/AL or 運動活動/AL or 身体活動/AL |
| #18 | 身体運動技術/TH |
| #19 | 余暇活動/TH or “leisure activities”/AL or (leisure/AL and activity/AL) or (leisure/AL and activities/AL) |
| #20 | 活動量計/TH or 活動量計/AL or “Fitness Trackers”/AL |
| #21 | 体育とトレーニング/TH or “physical training”/AL |
| #22 | 身体活動量の少ない生活/TH or “sedentary behaviour”/AL or “sedentary behaviour”/AL |
| #23 | #15 or #16 or #17 or #18 or #19 or #20 or #21 or #22 |
| #24 | #5 and #14 and #23 |
| #25 | 職業別集団/TH or worker/AL or労働者/AL or職場/AL or worksite/AL or workplace/AL or 雇用/TH or employ/AL or 職域/AL or 労働/TA or 仕事/TA or 就業/TA or 就労/TA |
| #26 | #24 and #25 |
| #27 | 欠勤/TH or absenteeism/AL or アブセンティーイズム/AL |
| #28 | 職務遂行能力/TH or 仕事のパフォーマンス/AL |
| #29 | プレゼンティーズム/TH or presenteeism/AL |
| #30 | 病気休暇/TH |
| #31 | 作業効率/TH or productivity/AL |
| #32 | 労働能力/AL or workability/AL or “work ability”/AL |
| #33 | 生活の質/TH |
| #34 | 労働衛生/TH |
| #35 | 作業効率/TH or Efficiency/AL |
| #36 | #27 or #28 or #29 or #30 or #31 or #32 or #33 or #34 or #35 |
| #37 | #26 and #36 |
| #38 | #37 and (PT=会議録を除く) |
