# Supplementary material for: Effectiveness of mHealth Interventions Aimed at Promoting Physical Activity and Reducing Sedentary Behavior on Work-Related Outcomes Among Workers: Systematic Review
Source: J Med Internet Res. 2026 May 7;28:e80540. doi: 10.2196/80540 (PMC13152228; doi:10.2196/80540)
Supplement: Multimedia Appendix 2 [file jmir-v28-e80540-s002.docx]

Multimedia Appendix 2: List of excluded studies along with reasons for exclusion.

Based on the original search conducted on September 23, 2023, a total of 238 full-text articles were excluded at the full-text screening stage.

List 1: Reasons for Exclusion Based on Participant Criteria (N=115).

| No | Article | Reason for exclusion |
| --- | --- | --- |
| 1 | Sahandi Far, M., Stolz, M., Fischer, J. M., Eickhoff, S. B., & Dukart, J. JTrack: a digital biomarker platform for remote monitoring of daily-life behaviour in health and disease. *Front. Public Health* **9,** 763621 (2021). | Participant　 criteria |
| 2 | Bustos, D. et al. Applicability of physiological monitoring systems within occupational groups: a systematic review. Sensors (Basel) **21,** 7249 (2021). | Participant　 criteria |
| 3 | Backes, A. et al. Advanced analytical methods to assess physical activity behavior using accelerometer time series: a scoping review. Scand. J. Med. Sci. Sports **32,** 18-44 (2022). | Participant　 criteria |
| 4 | Passos, J. et al. Wearables and Internet of Things (IoT) technologies for fitness assessment: a systematic review. Sensors (Basel) **21,** 5418 (2021). | Participant　 criteria |
| 5 | Meinke, A., Peters, R., Knols, R., Karlen, W., & Swanenburg, J. Exergaming using postural feedback from wearable sensors and exercise therapy to improve postural balance in people with nonspecific low back pain: protocol for a factorial pilot randomized controlled trial. JMIR Res. Protoc. **10,** e26982 (2021). | Participant　 criteria |
| 6 | Hung, N. T. et al. Wearable myoelectric interface enables high-dose, home-based training in severely impaired chronic stroke survivors. Ann. Clin. Transl. Neurol. **8,** 1895-1905 (2021). | Participant　 criteria |
| 7 | Eisenhauer, C. M. et al. Mobile health assisted self-monitoring is acceptable for supporting weight loss in rural men: a pragmatic randomized controlled feasibility trial. BMC Public Health **21,** 1568 (2021). | Participant　 criteria |
| 8 | Nickels, S. et al. Toward a mobile platform for real-world digital measurement of depression: user-centered design, data quality, and behavioral and clinical modeling. JMIR Ment. Health **8,** e27589 (2021). | Participant　 criteria |
| 9 | Eitzen, I., Renberg, J. & Faerevik, H. The use of wearable sensor technology to detect shock impacts in sports and occupational settings: a scoping review. Sensors (Basel) **21,** 4962 (2021). | Participant　 criteria |
| 10 | Tsou, M. M., Lung, S. C. & Cheng, C. Demonstrating the applicability of smartwatches in PM_2.5_ health impact assessment. Sensors (Basel) **21,** 4585 (2021). | Participant　 criteria |
| 11 | Resnick, D. et al. Promoting collaborative goal setting for cancer prevention among primary care patients through mHealth: mixed methods evaluation of a new app. JMIR Form. Res. **5,** e22510 (2021). | Participant　 criteria |
| 12 | Giurgiu, M. et al. Drivers of productivity: being physically active increases yet sedentary bouts and lack of sleep decrease work ability. Scand. J. Med. Sci. Sports **31,** 1921-1931 (2021). | Participant　 criteria |
| 13 | Márquez-Sánchez, S., Campero-Jurado, I., Robles-Camarillo, D., Rodríguez, S., Corchado-Rodríguez, J. M. BeSafe B2.0 smart multisensory platform for safety in workplaces. Sensors (Basel) **21,** 3372 (2021). | Participant　 criteria |
| 14 | Burns, S. P. et al. mHealth intervention applications for adults living with the effects of stroke: a scoping review. Arch. Rehabil. Res. Clin. Transl. **3,** 100095 (2020). | Participant　 criteria |
| 15 | Hinde, K., White, G. & Armstrong, N. Wearable devices suitable for monitoring twenty four hour heart rate variability in military populations. Sensors (Basel) **21,** 1061 (2021). | Participant　 criteria |
| 16 | Hui, D. & Mierzwinski-Urban, M. *Workplace electronic health promotion campaigns for tobacco smoking prevention or cessation [Internet]*. Ottawa (ON): Canadian Agency for Drugs and Technologies in Health (2021). | Participant　 criteria |
| 17 | Barth, J., Lohse, K. R., Konrad, J. D., Bland, M. D., & Lang, C. E. Sensor-based categorization of upper limb performance in daily life of persons with and without neurological upper limb deficits. Front. Rehabil. Sci. **2,** 741393 (2021). | Participant　 criteria |
| 18 | Bartholdy, C., Skou, S. T., Bliddal, H. & Henriksen, M. Changes in physical inactivity during supervised educational and exercise therapy in patients with knee osteoarthritis: a prospective cohort study. Knee **27,** 1848-1856 (2020). | Participant　 criteria |
| 19 | MacLean, B. L., MacLean, K., Stewart, I. B. & Hunt, A. P. Monitoring heat strain: the effect of sensor type and location on single-site and mean skin temperature during work in the heat. Int. Arch. Occup. Environ. Health **94,** 539-546 (2021). | Participant　 criteria |
| 20 | Truelove, S., Vanderloo, L. M., Tucker, P., Di Sebastiano, K. M., & Faulkner, G. The use of the Behaviour Change Wheel in the development of ParticipACTION's physical activity app. Prev. Med. Rep. **20,** 101224 (2020). | Participant　 criteria |
| 21 | Müssener, U. et al. Development of an intervention targeting multiple health behaviors among high school students: participatory design study using heuristic evaluation and usability testing. *JMIR Mhealth Uhealth* **8,** e17999 (2020). | Participant　 criteria |
| 22 | Schmidt-Kraepelin, M., Toussaint, P. A., Thiebes, S., Hamari, J., & Sunyaev, A. Archetypes of gamification: analysis of mHealth apps. *JMIR Mhealth Uhealth* **8,** e19280 (2020). | Participant　 criteria |
| 23 | Chen, X. S. et al. Association between behavioral phenotypes and response to a physical activity intervention using gamification and social incentives: secondary analysis of the STEP UP randomized clinical trial. PLoS One **15,** e0239288 (2020). | Participant　 criteria |
| 24 | Brower, J., LaBarge, M. C., White, L. & Mitchell, M. S. Examining responsiveness to an incentive-based mobile health app: longitudinal observational study. J. Med. Internet Res. **22,** e16797 (2020). | Participant　 criteria |
| 25 | Birnbaumer, P. et al. Absolute accelerometer-based intensity prescription compared to physiological variables in pregnant and nonpregnant women. Int. J. Environ. Res. Public Health **17,** 5651 (2020). | Participant　 criteria |
| 26 | Kumar, S. et al. Design, recruitment, and baseline characteristics of a virtual 1-year mental health study on behavioral data and health outcomes: observational study. JMIR Ment. Health **7,** e17075 (2020). | Participant　 criteria |
| 27 | Freak-Poli, R., Cumpston, M., Albarqouni, L., Clemes, S. A., & Peeters, A. Workplace pedometer interventions for increasing physical activity. Cochrane Database Syst. Rev. **7,** CD009209 (2020). | Participant　 criteria |
| 28 | Gupta, N. et al. The physical activity paradox revisited: a prospective study on compositional accelerometer data and long-term sickness absence. Int. J. Behav. Nutr. Phys. Act. **17,** 93 (2020). | Participant　 criteria |
| 29 | Murtagh, E. M. et al. Interventions outside the workplace for reducing sedentary behaviour in adults under 60 years of age. Cochrane Database Syst. Rev. **7,** CD012554 (2020). | Participant　 criteria |
| 30 | Mrklas, K. J. et al. Co-design in the development of a mobile health app for the management of knee osteoarthritis by patients and physicians: qualitative study. *JMIR Mhealth Uhealth* **8,** e17893 (2020). | Participant　 criteria |
| 31 | Haufe, S. et al. Employers with metabolic syndrome and increased depression/anxiety severity profit most from structured exercise intervention for work ability and quality of life. Front. Psychiatry **11,** 562 (2020). | Participant　 criteria |
| 32 | Harris, M. A. & Crone, D. Motivations and barriers to engagement with a technology-enabled community wide physical activity intervention. PLoS One **15,** e0232317 (2020). | Participant　 criteria |
| 33 | Janarthanan, V., Assad-Uz-Zaman, M., Rahman, M. H., McGonigle, E., & Wang, I. Design and development of a sensored glove for home-based rehabilitation. J. Hand Ther. **33,** 209-219 (2020). | Participant　 criteria |
| 34 | van Ekris, E. et al. Tracking of total sedentary time and sedentary patterns in youth: a pooled analysis using the International Children's Accelerometry Database (ICAD). Int. J. Behav. Nutr. Phys. Act. **17,** 65 (2020). | Participant　 criteria |
| 35 | Khundaqji, H., Hing, W., Furness, J. & Climstein, M. Smart shirts for monitoring physiological parameters: scoping review. *JMIR* *Mhealth Uhealth* **8,** e18092 (2020). | Participant　 criteria |
| 36 | Lines, R. L. J. et al. Cross-sectional and longitudinal comparisons of self-reported and device-assessed physical activity and sedentary behaviour. J. Sci. Med. Sport. **23,** 831-835 (2020). | Participant　 criteria |
| 37 | Chesser, S. A. et al. Exploring university age-friendliness using collaborative citizen science. Gerontologist **60,** 1527-1537 (2020). | Participant　 criteria |
| 38 | Stephenson, A. et al. Iterative four-phase development of a theory-based digital behaviour change intervention to reduce occupational sedentary behaviour. Digit. Health **6,** 2055207620913410 (2020). | Participant　 criteria |
| 39 | Hernández-Reyes, A. et al. Effectiveness of PUSH notifications from a mobile app for improving the body composition of overweight or obese women: a protocol of a three-armed randomized controlled trial. BMC Med. Inform. Decis. Mak. **20,** 40 (2020). | Participant　 criteria |
| 40 | De Carvalho, D. E. et al. Association of exposures to seated postures with immediate increases in back pain: a systematic review of studies with objectively measured sitting time. J. Manipulative Physiol. Ther. **43,** 1-12 (2020). | Participant　 criteria |
| 41 | Hassett, L. et al. Digitally enabled aged care and neurological rehabilitation to enhance outcomes with Activity and Mobility Using Technology (AMOUNT) in Australia: a randomised controlled trial. PLoS Med. **17,** e1003029 (2020). | Participant　 criteria |
| 42 | Mehta, S., Mehta, N., Tang, W. H. & Young, J. Cardiologists' perception of wearable device data in patients with heart failure. J. Gen. Intern. Med. **35,** 940-941 (2020). | Participant　 criteria |
| 43 | Taj, F., Klein, M. C. & Halteren, A. Digital health behavior change technology: bibliometric and scoping review of two decades of research. *JMIR Mhealth Uhealth* **7,** e13311 (2019). | Participant　 criteria |
| 44 | Stevenson, J. K. et al. eHealth interventions for people with chronic kidney disease. Cochrane Database Syst. Rev. **8,** CD012379 (2019). | Participant　 criteria |
| 45 | Boudet, G. et al. How to measure sedentary behavior at work? Front. Public Health **7,** 167 (2019). | Participant　 criteria |
| 46 | Millar, L. et al. Phase 3 diagnostic evaluation of a smart tablet serious game to identify autism in 760 children 3-5 years old in Sweden and the United Kingdom. BMJ Open **9,** e026226 (2019). | Participant　 criteria |
| 47 | Young, C. et al. Supporting engagement, adherence, and behavior change in online dietary interventions. J. Nutr. Educ. Behav. **51,** 719-739 (2019). | Participant　 criteria |
| 48 | Wilbur, R. E., Griffin, J. S., Sorensen, M., & Furberg, R. D. Establishing digital biomarkers for occupational health assessment in commercial salmon fishermen: protocol for a mixed-methods study. JMIR Res. Protoc. **7,** e10215 (2018). | Participant　 criteria |
| 49 | Friedl, K. E. Military applications of soldier physiological monitoring. J. Sci. Med. Sport **21,** 1147-1153 (2018). | Participant　 criteria |
| 50 | Wilde, L. J., Ward, G., Sewell, L., Müller, A. M., & Wark, P. A. Apps and wearables for monitoring physical activity and sedentary behaviour: a qualitative systematic review protocol on barriers and facilitators. Digit. Health **4,** 2055207618776454 (2018). | Participant　 criteria |
| 51 | Notley, S. R., Flouris, A. D. & Kenny, G. P. On the use of wearable physiological monitors to assess heat strain during occupational heat stress. Appl. Physiol. Nutr. Metab. **43,** 869-881 (2018). | Participant　 criteria |
| 52 | Kate, R. J., Swartz, A. M., Welch, W. A. & Strath, S. J. Comparative evaluation of features and techniques for identifying activity type and estimating energy cost from accelerometer data. Physiol. Meas. **37,** 360-379 (2016). | Participant　 criteria |
| 53 | Dicianno, B. E. et al. Perspectives on the evolution of mobile (mHealth) technologies and application to rehabilitation. Phys. Ther. **95,** 397-405 (2015). | Participant　 criteria |
| 54 | Rees, C. Recommendations for insulin dose calculator risk management. J. Diabetes Sci. Technol. **8,** 142-149 (2014). | Participant　 criteria |
| 55 | Milani, P. et al. Mobile smartphone applications for body position measurement in rehabilitation: a review of goniometric tools. PM R **6,** 1038-1043 (2014). | Participant　 criteria |
| 56 | Gao, L., Bourke, A. K. & Nelson, J. Evaluation of accelerometer based multi-sensor versus single-sensor activity recognition systems. Med. Eng. Phys. **36,** 779-785 (2014). | Participant　 criteria |
| 57 | Fronstin, P. Findings from the 2012 EBRI/MGA Consumer Engagement in Health Care Survey. EBRI Issue Brief **379,** 1-27 (2012). | Participant　 criteria |
| 58 | Choukou, M. A., He, E. & Moslenko, K. Feasibility of a virtual-reality-enabled at-home telerehabilitation program for stroke survivors: a case study. J. Pers. Med. **13,** 1230 (2023). | Participant　 criteria |
| 59 | Marwaa, M. N., Guidetti, S., Ytterberg, C. & Kristensen, H. K. Using experience-based co-design to develop mobile/tablet applications to support a person-centred and empowering stroke rehabilitation. Res. Involv. Engagem. **9,** 69 (2023). | Participant　 criteria |
| 60 | Moreno-Ligero, M., Moral-Munoz, J., Salazar, A. & Failde, I. mHealth intervention for improving pain, quality of life, and functional disability in patients with chronic pain: systematic review. *JMIR Mhealth Uhealth* **11,** e40844 (2023). | Participant　 criteria |
| 61 | Lee, Y., Lee, N. Y., Lim, H. J. & Sung, S. Weight reduction interventions using digital health for employees with obesity: a systematic review. Diabetes Metab. Syndr. Obes. **15,** 3121-3131 (2022). | Participant　 criteria |
| 62 | Martinsen, M., Zhou, Y., Dahlquist, E., Yan, J., & Kyprianidis, K. Positive climate effects when AR customer support simultaneous trains AI experts for the smart industries of the future. Appl. Energy **339,** 120988 (2023). | Participant　 criteria |
| 63 | De Bock, S. et al. An occupational shoulder exoskeleton reduces muscle activity and fatigue during overhead work. IEEE Trans. Biomed. Eng. **69,** 3008-3020 (2022). | Participant　 criteria |
| 64 | Ferry, T. et al. Outpatient subcutaneous antimicrobial therapy (OSCAT) as a measure to improve the quality and efficiency of healthcare delivery for patients with serious bacterial infections. Front. Med. (Lausanne) **7,** 585658 (2020). | Participant　 criteria |
| 65 | Zhang, L., Diraneyya, M. M., Ryu, J., Haas, C. T., & Abdel-Rahman, E. M. Jerk as an indicator of physical exertion and fatigue. Autom. Constr. **104,** 120-128 (2019). | Participant　 criteria |
| 66 | Nurse, C. A., Elstub, L. J., Volgyesi, P. & Zelik, K. E. How accurately can wearable sensors assess low back disorder risks during material handling? Exploring the fundamental capabilities and limitations of different sensor signals. Sensors (Basel) **23,** 2064 (2023). | Participant　 criteria |
| 67 | Kavuncuoğlu, E., Uzunhisaracikli, E., Barshan, B. & Ozdemir, A. T. Investigating the performance of wearable motion sensors on recognizing falls and daily activities via machine learning. Digit. Signal Process. **126,** 103365 (2022). | Participant　 criteria |
| 68 | Plewan, T., Mattig, B., Kretschmer, V. & Rinkenauer, G. Exploring the benefits and limitations of augmented reality for palletization. Appl. Ergon. **90,** 103250 (2021). | Participant　 criteria |
| 69 | Nassif, M. et al. Recruitment strategies of a decentralized randomized placebo-controlled clinical trial: the Canagliflozin Impact on Health Status, Quality of Life and Functional Status in Heart Failure (CHIEF-HF) trial. J. Card. Fail. **29,** 863-869 (2023). | Participant　 criteria |
| 70 | Ehrenfeld, J. M., Spickard, W. A. & Cutrer, W. B. Medical student contributions in the workplace: can we put a value on priceless? J. Med. Syst. **40,** 128 (2016). | Participant　 criteria |
| 71 | Sangalli, M. E. et al. Different HPMC viscosity grades as coating agents for an oral time and/or site-controlled delivery system: a study on process parameters and in vitro performances. Eur. J. Pharm. Sci. **22,** 469-476 (2004). | Participant　 criteria |
| 72 | Lambert, S. et al. Knowledge management for organisationally mobile public employees. Knowledge Management in Electronic Government: 4th IFIP International Working Conference, KMGov 2003, Rhodes, Greece, May 26-28, 2003 Proceedings 4, Springer Berlin Heidelberg (2003). | Participant　 criteria |
| 73 | Vasile, F., Vizziello, A., Brondino, N. & Savazzi, P. Stress state classification based on deep neural network and electrodermal activity modeling. Sensors (Basel) **23,** 2504 (2023). | Participant　 criteria |
| 74 | Arabshahi, M. et al. Review on sensing technology adoption in the construction industry. Sensors (Basel) **21,** 8307 (2021). | Participant　 criteria |
| 75 | Kini, R. Harrou, F., Madakyaru, M., Kadri, F., & Sun, Y. Efficient sitting posture recognition for wheelchair users: an unsupervised data-driven framework. IEEE Instrum. Meas. Mag. **26,** 37-43 (2023). | Participant　 criteria |
| 76 | Abd El-Aziz, M. et al. An effective data science technique for IoT-assisted healthcare monitoring system with a rapid adoption of cloud computing. Comput. Intell. Neurosci. **2022**, 7425846 (2022). | Participant　 criteria |
| 77 | Tam, A., Plotsker, E., Kim, M. & Thaller, S. R. Telemedicine for sports-related injuries. J. Craniofac. Surg. **32,** 1640-1643 (2021). | Participant　 criteria |
| 78 | Petsiuk, A. L. & Pearce, J. M. Low-cost open source ultrasound-sensing based navigational support for the visually impaired. Sensors (Basel) **19,** 3783 (2019). | Participant　 criteria |
| 79 | Kanoga, S., Hoshino, T. & Asoh, H. Semi-supervised style transfer mapping-based framework for sEMG-based pattern recognition with 1- or 2-DoF forearm motions. Biomed. Signal Process. Control **68,** 102817 (2021). | Participant　 criteria |
| 80 | Taheri-Garavand, A. et al. Automated in situ seed variety identification via deep learning: a case study in chickpea. Plants **10,** 1406 (2021). | Participant　 criteria |
| 81 | Liu, H.-P., Chuang, Y.-M., Liu, C.-H., Yang, P. C., & Fuh, C.-S. Precise measurement of physical activities and high-impact motion: feasibility of smart activity sensor system. IEEE Sens. J. **21,** 568-580 (2020). | Participant　 criteria |
| 82 | Aji, M. et al. A feasibility study of a mobile app to treat insomnia. Transl. Behav. Med. **11,** 604-612 (2021). | Participant　 criteria |
| 83 | Bohanec, M. et al. HeartMan DSS: a decision support system for self-management of congestive heart failure. Expert Syst. Appl. **186,** 115688 (2021). | Participant　 criteria |
| 84 | Allman-Farinelli, M. et al. The efficacy of electronic health interventions targeting improved sleep for achieving prevention of weight gain in adolescents and young to middle-aged adults: a systematic review. Obes. Rev. **21,** e13006 (2020). | Participant　 criteria |
| 85 | Marwaa, M. N., Guidetti, S., Ytterberg, C. & Kristensen, H. K. Use of mobile/tablet and web-based applications to support rehabilitation after stroke: a scoping review. J. Rehabil. Med. **54,** 452 (2022). | Participant　 criteria |
| 86 | Wongvibulsin, S. et al. Digital health interventions for cardiac rehabilitation: systematic literature review. *J. Med. Internet Res.* **23,** e18773 (2021). | Participant　 criteria |
| 87 | Donevant, S. B., Estrada, R. D., Culley, J. M., Habing, B., & Adams, S. A. Exploring app features with outcomes in mHealth studies involving chronic respiratory diseases, diabetes, and hypertension: a targeted exploration of the literature. J. Am. Med. Inform. Assoc. **25,** 1407-1418 (2018). | Participant　 criteria |
| 88 | Abdur Rahman, M. et al. A secure occupational therapy framework for monitoring cancer patients' quality of life. Sensors (Basel) **19**, 5258 (2019). | Participant　 criteria |
| 89 | Austad, H., Wiggen, O., Faerevik, H. & Seeberg, T. M. Towards a wearable sensor system for continuous occupational cold stress assessment. Ind. Health **56,** 228-240 (2018). | Participant　 criteria |
| 90 | Hwang, S. & Lee, S. Wristband-type wearable health devices to measure construction workers' physical demands. Autom. Constr. **83,** 330-340 (2017). | Participant　 criteria |
| 91 | Aksüt, G. & Eren, T. Selection of wearable sensors for health and safety use in the construction industry. J. Civ. Eng. Manag. **29,** 577-586 (2023). | Participant　 criteria |
| 92 | Duraisamy, A. & Subramaniam, M. Attack detection on IoT based smart cities using IDS based MANFIS classifier and secure data transmission using IRSA encryption. Wirel. Pers. Commun. **119**, 1913-1934 (2021). | Participant　 criteria |
| 93 | Sowiński, P. et al. Frugal heart rate correction method for scalable health and safety monitoring in construction sites. Sensors **23,** 6464 (2023). | Participant　 criteria |
| 94 | Ibrahim, A. A., Khan, M., Nnaji, C. & Koh, A. S. Assessing non-intrusive wearable devices for tracking core body temperature in hot working conditions. Appl. Sci. **13,** 6803 (2023). | Participant　 criteria |
| 95 | Cuesta-Morales, P. et al. VARSE: Android app for real-time acquisition and analysis of heart rate signals. Int. J. Med. Inform. **160,** 104692 (2022). | Participant　 criteria |
| 96 | Zadeh, A. et al. Predicting sports injuries with wearable technology and data analysis. Inf. Syst. Front. **23,** 1023-1037 (2021). | Participant　 criteria |
| 97 | Campero-Jurado, I., Márquez-Sánchez, S., Quintanar-Gómez, J., Rodríguez, S., & Corchado, J. M. Smart helmet 5.0 for industrial internet of things using artificial intelligence. Sensors (Basel) **20,** 6241 (2020). | Participant　 criteria |
| 98 | Soyka, F. & Simons, J. Improving the understanding of low frequency magnetic field exposure with augmented reality. Int. J. Environ. Res. Public Health **19,** 10564 (2022). | Participant　 criteria |
| 99 | Zakia, U. & Menon, C. Detecting safety anomalies in pHRI activities via force myography. Bioengineering **10,** 326 (2023). | Participant　 criteria |
| 100 | Sanchez-Iborra, R., G. Liaño, I., Simoes, C., Couñago, E., & Skarmeta, A. F. Tracking and monitoring system based on LoRa technology for lightweight boats. Electronics **8,** 15 (2018). | Participant　 criteria |
| 101 | Maman, Z. S. et al. A data analytic framework for physical fatigue management using wearable sensors. Expert Syst. Appl. **155,** 113405 (2020). | Participant　 criteria |
| 102 | Sagari, A. et al. Effect of occupation-based interventions in patients with haematopoietic malignancies undergoing chemotherapy: a pilot randomised controlled trial. Hong Kong J. Occup. Ther. **31,** 97-105 (2018). | Participant　 criteria |
| 103 | Ullah, M., Narayanan, A., Wolff, A. & Nardelli, P. H. J. Industrial energy management system: design of a conceptual framework using IoT and big data. IEEE Access **10,** 110557-110567 (2022). | Participant　 criteria |
| 104 | Yeni, S., Cagiltay, K. & Karasu, N. Usability investigation of an educational mobile application for individuals with intellectual disabilities. Univers. Access Inf. Soc. **19,** 619-632 (2020). | Participant　 criteria |
| 105 | Lopez, M. A., Terron, S., Lombardo, J. M. & Gonzalez-Crespo, R. Towards a solution to create, test and publish mixed reality experiences for occupational safety and health learning: Training-MR. *Int. J. Interact. Multimed. Artif. Intell.* (2021). | Participant　 criteria |
| 106 | Waqar, A. et al. Assessment of Challenges to the Adoption of IOT for the Safety Management of Small Construction Projects in Malaysia: Structural Equation Modeling Approach. *Appl.* *Sci.* **13**(5)**,** 3340 (2023). | Participant　 criteria |
| 107 | Mekruksavanich, S. & Jitpattanakul, A. Automatic recognition of construction worker activities using deep learning approaches and wearable inertial sensors. Intell. Autom. Soft Comput. **36,** 2 (2023). | Participant　 criteria |
| 108 | Asensio-Cuesta, S. et al. Smartphone sensors for monitoring cancer-related quality of life: app design, EORTC QLQ-C30 mapping and feasibility study in healthy subjects. Int. J. Environ. Res. Public Health **16,** 461 (2019). | Participant　 criteria |
| 109 | Kalanadhabhatta, M., Rahman, T. & Ganesan, D. Effect of sleep and biobehavioral patterns on multidimensional cognitive performance: longitudinal, in-the-wild study. *J. Med. Internet Res.* **23,** e23936 (2021). | Participant　 criteria |
| 110 | Carpintero-Rubio, C. et al. Perception of musculoskeletal pain in the state of confinement: associated factors. Rev. Lat-Am. Enfermagem. **29,** e3454 (2021). | Participant　 criteria |
| 111 | Birney, A. J., Gunn, R., Russell, J. K. & Ary, D. V. MoodHacker mobile web app with email for adults to self-manage mild-to-moderate depression: randomized controlled trial. *JMIR Mhealth Uhealth***4,** e4231 (2016). | Participant　 criteria |
| 112 | Kiehl, Z. A., Durkee, K. T., Halverson, K., Christensen, J., & Hellstern, G. Transforming work through human sensing: a confined space monitoring application. Struct. Health Monit. **19,** 186-201 (2020). | Participant　 criteria |
| 113 | Antwi-Afari, M. F. et al. Machine learning-based identification and classification of physical fatigue levels: a novel method based on a wearable insole device. Int. J. Ind. Ergon. **93,** 103404 (2023). | Participant　 criteria |
| 114 | Calvetti, D., Mêda, P., Chichorro Gonçalves, M., & Sousa, H. Worker 4.0: The Future of Sensored Construction Sites. *Buildings* **10,** 169 (2020). | Participant　 criteria |
| 115 | Irvine, A. B., et al. Mobile-Web app to self-manage low back pain: randomized controlled trial. *J. Med. Internet Res.* **17,** e1 (2015). | Participant　 criteria |

List 2: Reasons for Exclusion Based on Intervention Criteria (N=72).

| No | Article | Reason for exclusion |
| --- | --- | --- |
| 1 | Smith-MacDonald, L. et al. The experience of key stakeholders during the implementation and use of trauma therapy via digital health for military, veteran, and public safety personnel: qualitative thematic analysis. *JMIR Form Res.* **5,** e26369 (2021). | Intervention criteria |
| 2 | Markopoulos, P., Shen, X., Wang, Q. & Timmermans, A. Neckio: motivating neck exercises in computer workers. Sensors (Basel) **20,** 4928 (2020). | Intervention criteria |
| 3 | Wong, K. H., Bayarsaikhan, S., Levine, B. A. & Mun, S. K. Prototype of a military medic smartphone medical graphical user interface for use by medics in deployed environments. Mil. Med. **185,** S536-S543 (2020). | Intervention criteria |
| 4 | Razjouyan, J. et al. Wellbuilt for wellbeing: controlling relative humidity in the workplace matters for our health. Indoor Air **30,** 167-179 (2020). | Intervention criteria |
| 5 | Lucas, B. et al. Sex differences in heart rate responses to occupational stress. Stress **23,** 13-18 (2020). | Intervention criteria |
| 6 | Edwardson, C. L. et al. Effectiveness of an intervention for reducing sitting time and improving health in office workers: three arm cluster randomised controlled trial. BMJ **378,** e069288 (2022). | Intervention criteria |
| 7 | Collins, D. A. J. et al. A pilot evaluation of a smartphone application for workplace depression. Int. J. Environ. Res. Public Health **17,** 6753 (2020). | Intervention criteria |
| 8 | Massaroni, C. et al. Contact-based methods for measuring respiratory rate. Sensors (Basel) **19,** 908 (2019). | Intervention criteria |
| 9 | Sugawara, J. et al. Maternity Log study: a longitudinal lifelog monitoring and multiomics analysis for the early prediction of complicated pregnancy. BMJ Open **9,** e025939 (2019). | Intervention criteria |
| 10 | Fan, Y. C. & Wen, C. Y. A virtual reality soldier simulator with body area networks for team training. Sensors (Basel) **19,** 451 (2019). | Intervention criteria |
| 11 | Oliver, M., Teruel, M. A., Molina, J. P., Romero-Ayuso, D., & González, P. Ambient intelligence environment for home cognitive telerehabilitation. Sensors (Basel) **18,** 3671 (2018). | Intervention criteria |
| 12 | Leese, J. et al. Using physical activity trackers in arthritis self-management: a qualitative study of patient and rehabilitation professional perspectives. Arthritis Care Res. (Hoboken) **71,** 227-236 (2019). | Intervention criteria |
| 13 | Thompson, J. F., Severson, R. L. & Rosecrance, J. C. Occupational physical activity in brewery and office workers. J. Occup. Environ. Hyg. **15**, 686-699 (2018). | Intervention criteria |
| 14 | Ropponen, A., Härmä, M., Bergbom, B., Nätti, J., & Sallinen, M. The vicious circle of working hours, sleep, and recovery in expert work. Int. J. Environ. Res. Public Health **15,** 1361 (2018). | Intervention criteria |
| 15 | Lee, S. I. et al. Enabling stroke rehabilitation in home and community settings: a wearable sensor-based approach for upper-limb motor training. IEEE J. Transl. Eng. Health Med. **6**, 2100411 (2018). | Intervention criteria |
| 16 | Soh, J. Y. et al. Development and validation of a multidisciplinary mobile care system for patients with advanced gastrointestinal cancer: interventional observation study. *JMIR Mhealth Uhealth* **6,** e115 (2018). | Intervention criteria |
| 17 | Marchesseault, E. R. et al. Head impacts and cognitive performance in men's lacrosse. Phys. Sportsmed. **46,** 324-330 (2018). | Intervention criteria |
| 18 | Emmerson, K. B., Harding, K. E., Lockwood, K. J. & Taylor, N. F. Home exercise programs supported by video and automated reminders for patients with stroke: a qualitative analysis. Aust. Occup. Ther. J. **65,** 187-197 (2018). | Intervention criteria |
| 19 | Pancardo, P., Acosta, F. D., Hernández-Nolasco, J. A., Wister, M. A., & López-de-Ipiña, D. Real-time personalized monitoring to estimate occupational heat stress in ambient assisted working. Sensors (Basel) **15,** 16956-16980 (2015). | Intervention criteria |
| 20 | Morris, M. E. et al. Mobile therapy: case study evaluations of a cell phone application for emotional self-awareness. *J. Med. Internet Res.* **12,** e10 (2010). | Intervention criteria |
| 21 | Coca, A. et al. Physiological monitoring in firefighter ensembles: wearable plethysmographic sensor vest versus standard equipment. J. Occup. Environ. Hyg. **7,** 109-114 (2010). | Intervention criteria |
| 22 | Krafft, J. et al. A tablet-based app to support nursing home staff in delivering an individualized cognitive and physical exercise program for individuals with dementia: mixed methods usability study. JMIR Aging **6,** e46480 (2023). | Intervention criteria |
| 23 | Bayartai, M. E. et al. Role of the interaction between lumbar kinematics and accelerometer-measured physical activity in bodily pain, physical functioning and work ability among health care workers with low back pain. J. Electromyogr. Kinesiol. **69,** 102744 (2023). | Intervention criteria |
| 24 | Li, G., Yuan, C., Kamarthi, S., Moghaddam, M., & Jin, X. Data science skills and domain knowledge requirements in the manufacturing industry: a gap analysis. J. Manuf. Syst. **60,** 692-706 (2021). | Intervention criteria |
| 25 | Antwi-Afari, M. F. et al. Deep learning-based networks for automated recognition and classification of awkward working postures in construction using wearable insole sensor data. Autom. Constr. **136,** 104181 (2022). | Intervention criteria |
| 26 | Umer, W. et al. Physical exertion modeling for construction tasks using combined cardiorespiratory and thermoregulatory measures. Autom. Constr. **112,** 103079 (2020). | Intervention criteria |
| 27 | Stucky, B. et al. Validation of Fitbit Charge 2 sleep and heart rate estimates against polysomnographic measures in shift workers: naturalistic study. *J. Med. Internet Res.* **23,** e26476 (2021). | Intervention criteria |
| 28 | Xing, X. et al. Effects of physical fatigue on the induction of mental fatigue of construction workers: a pilot study based on a neurophysiological approach. Autom. Constr. **120,** 103381 (2020). | Intervention criteria |
| 29 | Zhao, U. & Obonyo, E. Applying incremental deep neural networks-based posture recognition model for ergonomics risk assessment in construction. Adv. Eng. Inform. **50,** 101374 (2021). | Intervention criteria |
| 30 | Antwi-Afari, M. F., Li, H., Umer, W., Yu, Y. & Xing, X. Construction activity recognition and ergonomic risk assessment using a wearable insole pressure system. J. Constr. Eng. Manag. **146,** 04020077 (2020). | Intervention criteria |
| 31 | Li, S., Jiang, Y., Sun, C., Guo, K. & Wang, X. An investigation on the influence of operation experience on virtual hazard perception using wearable eye tracking technology. Sensors *(Basel)* **22,** 5115 (2022). | Intervention criteria |
| 32 | Pérez, C. T., Salling, S. & Wandahl, S. Five guidelines for adopting smartwatches in construction: a novel approach for understanding workers' efficiency based on travelled distances and locations. Sustainability **14,** 8875 (2022). | Intervention criteria |
| 33 | Du, T., Iwakiri, K., Sotoyama, M., Tokizawa, K. & Oyama, F. Relationship between using tables, chairs, and computers and improper postures when doing VDT work in work from home. Ind. Health **60,** 307-318 (2022). | Intervention criteria |
| 34 | Isunju, J. B. et al. Awareness of hepatitis B post-exposure prophylaxis among healthcare providers in Wakiso district, Central Uganda. PLoS One **17,** e0270181 (2022). | Intervention criteria |
| 35 | O'Donovan, J., O'Donovan, C., Kuhn, I., Sachs, SE. & Winters, N. Ongoing training of community health workers in low-income and middle-income countries: a systematic scoping review of the literature. BMJ Open **8,** e021467 (2018). | Intervention criteria |
| 36 | Khamaisi, R., Brunzini, A., Grandi, F., Peruzzini, M & Pellicciari, M. UX assessment strategy to identify potential stressful conditions for workers. Robot. Comput.-Integr. Manuf. **78,** 102403 (2022). | Intervention criteria |
| 37 | Lang, A. et al. The impact of an electronic patient bedside observation and handover system on clinical practice: mixed-methods evaluation. JMIR Med. Inform. **7,** e11678 (2019). | Intervention criteria |
| 38 | Al-Hussaini, I. & Mitchell, C. S. SeizFt: interpretable machine learning for seizure detection using wearables. Bioengineering **10,** 918 (2023). | Intervention criteria |
| 39 | Tavares, C. et al. Smart office chair for working conditions optimization. IEEE Access **11,** 50497-50509 (2023). | Intervention criteria |
| 40 | Koşar, E. & Barshan, B. A new CNN-LSTM architecture for activity recognition employing wearable motion sensor data: enabling diverse feature extraction. Eng. Appl. Artif. Intell. **124,** 106529 (2023). | Intervention criteria |
| 41 | Venkatachalam, K. et al. Bimodal HAR—an efficient approach to human activity analysis and recognition using bimodal hybrid classifiers. Inf. Sci. **628,** 542-557 (2023). | Intervention criteria |
| 42 | Black, N. J., Cheng, T. Y. & Arruda, A. G. Characterizing the connection between swine production sites by personnel movements using a mobile application-based geofencing platform. Prev. Vet. Med. **208,** 105753 (2022). | Intervention criteria |
| 43 | Esfahani, A. H., Dyka, Z., Ortmann, S. & Langendorfer, P. Impact of data preparation in freezing of gait detection using feature-less recurrent neural network. IEEE Access **9,** 138120-138131 (2021). | Intervention criteria |
| 44 | Chow, J. S. F. et al. Teleworking from home experiences during the COVID-19 pandemic among public health workers (TelEx COVID-19 study). BMC Public Health **22,** 674 (2022). | Intervention criteria |
| 45 | Fowler, L. A., Hirsh, EL., Klinefelter, Z., Sulzbach, M. & Britt, TW. Objective assessment of sleep and fatigue risk in emergency medicine physicians. Acad. Emerg. Med. **30,** 166-171 (2023). | Intervention criteria |
| 46 | Alshamsi, A., Pianesi, F., Lepri, B., Pentland, A. & Rahwan, I. Beyond contagion: reality mining reveals complex patterns of social influence. PLoS ONE **10,** e0135740 (2015). | Intervention criteria |
| 47 | Halim, Z. & Rehan, M. On identification of driving-induced stress using electroencephalogram signals: a framework based on wearable safety-critical scheme and machine learning. Inf. Fusion **53,** 66-79 (2020). | Intervention criteria |
| 48 | Yang, L. et al. Towards smart work clothing for automatic risk assessment of physical workload. IEEE Access **6,** 40059-40072 (2018). | Intervention criteria |
| 49 | Marín-Farrona, M. J. et al. Influence of non-occupational physical activity on burnout syndrome, job satisfaction, stress and recovery in fitness professionals. Int. J. Environ. Res. Public Health **18,** 9489 (2021). | Intervention criteria |
| 50 | Huberty, J. L., Espel-Huynh, H. M., Neher, T. L. & Puzia, M. E. Testing the pragmatic effectiveness of a consumer-based mindfulness mobile app in the workplace: randomized controlled trial. *JMIR Mhealth Uhealth* **10,** e38903 (2022). | Intervention criteria |
| 51 | Lee, W., Lin, K.-Y., Seto, E. & Migliaccio, G. C. Wearable sensors for monitoring on-duty and off-duty worker physiological status and activities in construction. Autom. Constr. **83,** 341-353 (2017). | Intervention criteria |
| 52 | Valdivia, S. et al. Development and evaluation of two posture-tracking user interfaces for occupational health care. Adv. Mech. Eng. **10,** 1687814018769489 (2018). | Intervention criteria |
| 53 | Deady, M. et al. Preliminary effectiveness of a smartphone app to reduce depressive symptoms in the workplace: feasibility and acceptability study. *JMIR Mhealth Uhealth* **6,** e11661 (2018). | Intervention criteria |
| 54 | Onofrejova, D., Balazikova, M., Glatz, J., Kotianova, Z. & Vaskovicova, K. Ergonomic assessment of physical load in Slovak industry using wearable technologies. Appl. Sci. **12,** 3607 (2022). | Intervention criteria |
| 55 | Bagheri, M., Siekkinen, M. & Nurminen, J. K. Cloud-based pedestrian road-safety with situation-adaptive energy-efficient communication. IEEE Intell. Transp. Syst. Mag. **8,** 45-62 (2016). | Intervention criteria |
| 56 | Lindquist, A., Johansson, PE., Petersson, GI., Saveman, BI. & Nilsson, GC. The use of the Personal Digital Assistant (PDA) among personnel and students in health care: a review. J. Med. Internet Res. **10,** e31 (2008). | Intervention criteria |
| 57 | Vallières, F., McAuliffe, E., van Bavel, B., Wall, PJ. & Trye, A. There's no app for that: assessing the impact of mHealth on the supervision, motivation, engagement, and satisfaction of community health workers in Sierra Leone. Ann. Glob. Health **82,** 936-949 (2016). | Intervention criteria |
| 58 | Kimball, J. P., Inan, OT., Convertino, VA., Cardin, S. & Sawka, MN. Wearable sensors and machine learning for hypovolemia problems in occupational, military and sports medicine: physiological basis, hardware and algorithms. Sensors (Basel) **22,** 442 (2022). | Intervention criteria |
| 59 | Donisi, L. et al. Wearable sensors and artificial intelligence for physical ergonomics: a systematic review of literature. Diagnostics **12,** 3048 (2022). | Intervention criteria |
| 60 | Heber, E. et al. Efficacy and cost-effectiveness of a web-based and mobile stress-management intervention for employees: design of a randomized controlled trial. BMC Public Health **13,** 655 (2013). | Intervention criteria |
| 61 | Xu, H., Eley, R., Kynoch, K. & Tuckett, A. Effects of mobile mindfulness on emergency department work stress: a randomised controlled trial. Emerg. Med. Australas. **34,** 176-185 (2022). | Intervention criteria |
| 62 | David, D., Lin, SY., Groom, LL., Ford, A. & Brody, AA. Aliviado mobile app for hospice providers: a usability study. J. Pain Symptom Manage. **63,** e37-e45 (2022). | Intervention criteria |
| 63 | Mardonova, M. & Choi, Y. Review of wearable device technology and its applications to the mining industry. Energies **11,** 547 (2018). | Intervention criteria |
| 64 | Van Beukering, M. et al. Evaluation of a blended care programme for caregivers and working pregnant women to prevent adverse pregnancy outcomes: an intervention study. Occup. Environ. Med. **78,** 809-817 (2021). | Intervention criteria |
| 65 | Imtiaz, S. A., Krishnaiah, S., Yadav, SK., Bharath, B. & Ramani, RV. Benefits of an android based tablet application in primary screening for eye diseases in a rural population, India. J. Med. Syst. **41,** 49 (2017). | Intervention criteria |
| 66 | Rubiano, L. et al. Adaptation and performance of a mobile application for early detection of cutaneous leishmaniasis. PLoS Negl. Trop. Dis. **15,** e0008989 (2021). | Intervention criteria |
| 67 | Elder, E., Johnston, AN., Byrne, JH., Wallis, M. & Crilly, J. Core components of a staff wellness strategy in emergency departments: a clinician‐informed nominal group study. Emerg. Med. Australas. **33,** 25-33 (2021). | Intervention criteria |
| 68 | Izumi, K. et al. Unobtrusive sensing technology for quantifying stress and well-being using pulse, speech, body motion, and electrodermal data in a workplace setting: study concept and design. Front. Psychiatry **12,** 611243 (2021). | Intervention criteria |
| 69 | Marks, A. N., Sol, JA., Domitrovich, JW., West, MR. & Ruby, BC. Total energy intake and self-selected macronutrient distribution during wildland fire suppression. Wilderness Environ. Med. **31,** 188-196 (2020). | Intervention criteria |
| 70 | Tsai, M.-K., Yang, J.-B. & Lin, C.-Y. Integrating wireless and speech technologies for synchronous on-site data collection. Autom. Constr. **16,** 378-391 (2007). | Intervention criteria |
| 71 | Lin, K.-Y. et al. A user-centered information and communication technology (ICT) tool to improve safety inspections. Autom. Constr. **48,** 53-63 (2014). | Intervention criteria |
| 72 | Hojati, Z., Goudarzi, F., Hasanvand, S., Galehdar, N. & Birjandi, M. The impact of training chemotherapy safety standards with a smartphone application on the knowledge, attitude, and performance of nurses. BMC Nurs. **22,** 43 (2023). | Intervention criteria |

List 3: Reasons for Exclusion Based on Outcome Criteria (N=48).

| No | Article | Reason for exclusion |
| --- | --- | --- |
| 1 | Enayati, M. et al. Incorporating RTLS-based spatiotemporal information in studying physical activities of clinical staff. *Annu. Int. Conf. IEEE Eng. Med. Biol. Soc*. 2386-2391 (2021). | Outcome criteria |
| 2 | Ben-David, R. et al. Resident physicians physical activity during on-call shifts: smartphone-based assessment. Occup. Med. (Lond.) **72,** 105-109 (2022). | Outcome criteria |
| 3 | Garza, J. L., Wu, Z. H., Singh, M. & Cherniack, M. G. Comparison of the wrist-worn Fitbit Charge 2 and the waist-worn Actigraph GTX3 for measuring steps taken in occupational settings. Ann. Work Expo. Health **66,** 281-284 (2022). | Outcome criteria |
| 4 | Wang, X. et al. Monitoring work-related physical activity and estimating lower-limb loading: a proof-of-concept study. BMC Musculoskelet. Disord. **22,** 552 (2021). | Outcome criteria |
| 5 | Syrjälä, M. B., Fharm, E., Dempsey, PC., Nordendahl, M. & Wennberg, P. Reducing occupational sitting time in adults with type 2 diabetes: qualitative experiences of an office-adapted mHealth intervention. Diabet. Med. **38,** e14514 (2021). | Outcome criteria |
| 6 | Huang, Y. et al. Using Internet of Things to reduce office workers' sedentary behavior: intervention development applying the Behavior Change Wheel and human-centered design approach. *JMIR Mhealth Uhealth* **8,** e17914 (2020). | Outcome criteria |
| 7 | Nicolson, G. H., Hayes, C. B. & Darker, C. D. A cluster-randomised crossover pilot feasibility study of a multicomponent intervention to reduce occupational sedentary behaviour in professional male employees. Int. J. Environ. Res. Public Health **18,** 9292 (2021). | Outcome criteria |
| 8 | Stephenson, A. et al. The "Worktivity" mHealth intervention to reduce sedentary behaviour in the workplace: a feasibility cluster randomised controlled pilot study. BMC Public Health **21,** 1416 (2021). | Outcome criteria |
| 9 | Halse, R. E. et al. Improving nutrition and activity behaviors using digital technology and tailored feedback: protocol for the Tailored Diet and Activity (ToDAy) randomized controlled trial. JMIR Res. Protoc. **8,** e12782 (2019). | Outcome criteria |
| 10 | Lawrie, S. et al. Evaluation of a smartwatch-based intervention providing feedback of daily activity within a research-naive stroke ward: a pilot randomised controlled trial. Pilot Feasibility Stud. **4,** 157 (2018). | Outcome criteria |
| 11 | Aschbrenner, K. A. et al. Peer support and mobile health technology targeting obesity-related cardiovascular risk in young adults with serious mental illness: protocol for a randomized controlled trial. Contemp. Clin. Trials **74,** 97-106 (2018). | Outcome criteria |
| 12 | Olsen, H. M., Brown, W. J., Kolbe-Alexander, T. & Burton, N. W. A brief self-directed intervention to reduce office employees' sedentary behavior in a flexible workplace. J. Occup. Environ. Med. **60,** 954-959 (2018). | Outcome criteria |
| 13 | Risica, P. M. et al. A multi-level intervention in worksites to increase fruit and vegetable access and intake: rationale, design and methods of the 'Good to Go' cluster randomized trial. Contemp. Clin. Trials **65,** 87-98 (2018). | Outcome criteria |
| 14 | Guitar, N. A., MacDougall, A., Connelly, D. M. & Knight, E. Fitbit activity trackers interrupt workplace sedentary behavior: a new application. Workplace Health Saf. **66,** 218-222 (2018). | Outcome criteria |
| 15 | Gilson, N. D. et al. The impact of an m-Health financial incentives program on the physical activity and diet of Australian truck drivers. BMC Public Health **17,** 467 (2017). | Outcome criteria |
| 16 | van Drongelen, A., Boot, CR., Hlobil, H., Smid, T. & van der Beek, AJ. Process evaluation of a tailored mobile health intervention aiming to reduce fatigue in airline pilots. BMC Public Health. **16,** 894 (2016). | Outcome criteria |
| 17 | van Drongelen, A., van der Beek AJ., Hlobil H., Smid T. & Boot CR. Development and evaluation of an intervention aiming to reduce fatigue in airline pilots: design of a randomised controlled trial. BMC Public Health **13,** 776 (2013). | Outcome criteria |
| 18 | Sevic, A. et al. Effectiveness of eHealth interventions targeting employee health behaviors: systematic review. J. Med. Internet Res. **25,** e38307 (2023). | Outcome criteria |
| 19 | Huang, Y., Benford, S., Li, B., Price, D. & Blake, H. Feasibility and acceptability of an Internet of Things-enabled sedentary behavior intervention: mixed methods study. J. Med. Internet Res. **25,** e43502 (2023). | Outcome criteria |
| 20 | Edwards, N. A., Talarico MK, Chaudhari A, Mansfield CJ & Oñate J. Use of accelerometers and inertial measurement units to quantify movement of tactical athletes: a systematic review. Appl. Ergon. **109,** 103991 (2023). | Outcome criteria |
| 21 | Monnaatsie, M., Biddle, S. J. H. & Kolbe-Alexander, T. The feasibility of a text-messaging intervention promoting physical activity in shift workers: a process evaluation. Int. J. Environ. Res. Public Health **20,** 3260 (2023). | Outcome criteria |
| 22 | Wilson, D., Driller, M. W., Johnston, B. & Gill, N. D. A contactless app-based intervention to improve health behaviors in airline pilots: a randomized trial. Am. J. Prev. Med. **64,** 666-676 (2023). | Outcome criteria |
| 23 | Signorini, G., Scurati R, D'Angelo C, Rigon M & Invernizzi PL. Enhancing motivation and psychological wellbeing in the workplace through conscious physical activity: suggestions from a qualitative study examining workers' experience. Front. Psychol. **13,** 1006876 (2022). | Outcome criteria |
| 24 | Sikalidis, A. K. et al. Capacity strengthening undertaking-farm organized response of workers against risk for diabetes: (C.S.U.-F.O.R.W.A.R.D. with Cal Poly)—a concept approach to tackling diabetes in vulnerable and underserved farmworkers in California. Sensors (Basel) **22,** 8299 (2022). | Outcome criteria |
| 25 | Bayerle, P. et al. Effectiveness of wearable devices as a support strategy for maintaining physical activity after a structured exercise intervention for employees with metabolic syndrome: a randomized controlled trial. BMC Sports Sci. Med. Rehabil. **14,** 24 (2022). | Outcome criteria |
| 26 | Larsen, R. T. et al. Effectiveness of physical activity monitors in adults: systematic review and meta-analysis. BMJ **376,** e068047 (2022). | Outcome criteria |
| 27 | Gancitano, G. et al. HRV in active-duty special forces and public order military personnel. Sustainability **13,** 3867 (2021). | Outcome criteria |
| 28 | Park, S., Seong, S., Ahn, Y. & Kim, H. Real-time fatigue evaluation using ecological momentary assessment and smartwatch data: an observational field study on construction workers. J. Manage. Eng. **39,** 04023008 (2023). | Outcome criteria |
| 29 | Zulauf-Czaja, A. et al. On the way home: a BCI-FES hand therapy self-managed by sub-acute SCI participants and their caregivers: a usability study. J. Neuroeng. Rehabil. **18,** 44 (2021). | Outcome criteria |
| 30 | Murphree, D. H. et al. Measuring the impact of ambulatory red blood cell transfusion on home functional status: study protocol for a pilot randomized controlled trial. Trials **18,** 153 (2017). | Outcome criteria |
| 31 | Patterson, P. D. et al. Fatigue mitigation with SleepTrackTXT2 in air medical emergency care systems: study protocol for a randomized controlled trial. Trials **18,** 254 (2017). | Outcome criteria |
| 32 | Xing, X. et al. A multicomponent and neurophysiological intervention for the emotional and mental states of high-altitude construction workers. Autom. Constr. **105,** 102836 (2019). | Outcome criteria |
| 33 | Brandt, M. et al. Effects of a participatory ergonomics intervention with wearable technical measurements of physical workload in the construction industry: cluster randomized controlled trial. J. Med. Internet Res. **20,** e10272 (2018). | Outcome criteria |
| 34 | Shrestha, N. et al. Effectiveness of interventions for reducing non-occupational sedentary behaviour in adults and older adults: a systematic review and meta-analysis. Br. J. Sports Med. **53,** 1206-1213 (2019). | Outcome criteria |
| 35 | Barati Jozan, M. M., Ghorbani, BD., Khalid, MS., Lotfata, A. & Tabesh, H. Impact assessment of e-trainings in occupational safety and health: a literature review. BMC Public Health **23,** 1187 (2023). | Outcome criteria |
| 36 | Compernolle, S. et al. Effectiveness of interventions using self-monitoring to reduce sedentary behavior in adults: a systematic review and meta-analysis. Int. J. Behav. Nutr. Phys. Act. **16,** 63 (2019). | Outcome criteria |
| 37 | Subhan, F. et al. AI-enabled wearable medical internet of things in healthcare system: a survey. Appl. Sci. **13,** 1394 (2023). | Outcome criteria |
| 38 | Simons, D. et al. Effect and process evaluation of a smartphone app to promote an active lifestyle in lower educated working young adults: cluster randomized controlled trial. *JMIR Mhealth Uhealth* **6,** e10003 (2018). | Outcome criteria |
| 39 | Hilty, D. M. et al. Findings and guidelines on provider technology, fatigue, and well-being: scoping review. J. Med. Internet Res. **24,** e34451 (2022). | Outcome criteria |
| 40 | Lemos, J., Gaspar, P. D. & Lima, T. M. Environmental risk assessment and management in industry 4.0: a review of technologies and trends. Machines **10,** 702 (2022). | Outcome criteria |
| 41 | Nahm, J. W. & Shin, Y. J. Effects of mobile-based exercise intervention on health indices by the comparison of personal training time in male workers. Ethiop. J. Health Dev. **34,** 10-17 (2020). | Outcome criteria |
| 42 | Xie, X. & Chang, Z. Intelligent wearable occupational health safety assurance system of power operation. J. Med. Syst. **43,** 16 (2019). | Outcome criteria |
| 43 | Brørs, G. et al. Modes of e-Health delivery in secondary prevention programmes for patients with coronary artery disease: a systematic review. BMC Health Serv. Res. **19,** 364 (2019). | Outcome criteria |
| 44 | Biddle, S. J. H. et al. Reducing sitting at work: process evaluation of the SMArT Work (Stand More At Work) intervention. Trials **21,** 403 (2020). | Outcome criteria |
| 45 | Lee, J. H. et al. Effectiveness of an application-based neck exercise as a pain management tool for office workers with chronic neck pain and functional disability: a pilot randomized trial. Eur. J. Integr. Med. **12,** 87-92 (2017). | Outcome criteria |
| 46 | Guan, Y. Y., Chen C. & Yoong, J. S. Using financial incentives to motivate stair use in a workplace setting: results from a randomized controlled trial. Value Health **18,** A568 (2015). | Outcome criteria |
| 47 | Yamamoto, K. et al. Identifying characteristics of indicators of sedentary behavior using objective measurements. J. Occup. Health **62,** e12089 (2020). | Outcome criteria |
| 48 | Arrogi, A., Boen, F. & Seghers, J. Validation of a smart chair and corresponding smartphone app as an objective measure of desk-based sitting. J. Occup. Health **61,** 121-127 (2019). | Outcome criteria |

List 4: Reasons for Exclusion Based on Not original article (N=3).

| No | Article | Reason 　　for exclusion |
| --- | --- | --- |
| 1 | Deng, H. et al. Digital interventions for social participation in adults with long-term physical conditions: a systematic review. *J. Med. Syst.* **47,** 26 (2023). | Not 　original article |
| 2 | Turesson, C., Liedberg, G., Vixner, L., Lofgren, M. & Björk, M. Evidence-based digital support during 1 year after an interdisciplinary pain rehabilitation programme for persons with chronic musculoskeletal pain to facilitate a sustainable return to work: a study protocol for a registry-based multicentre randomised controlled trial. *BMJ Open* **12,** e060452 (2022). | Not 　original article |
| 3 | Koriyama, S. & Sawada, S. Health and productivity management and physical activity at the work-site. *Job Stress Res*. **28,** 241-247 (2021). [in Japanese] | Not 　original article |

Based on the updated search conducted on September 12, 2025, a total of 31 full-text articles were excluded at the full-text screening stage.

List 1: Reasons for Exclusion Based on Participant Criteria (N=6).

| No | Article | Reason for exclusion |
| --- | --- | --- |
| 1 | Javed, A. et al. Personalized digital behaviour interventions increase short-term physical activity: a randomized controlled crossover trial substudy of the MyHeart Counts Cardiovascular Health Study. *Eur. Heart J. Digit. Health* **4***,* 411–419 (2023). | Participant　 criteria |
| 2 | Strach, M., Różanowski, K., Pietrucha, J. & Lewandowski, J. Analysis of the functionality of a mobile network of sensors in a construction project supervision system based on unmanned aerial vehicles. *Sustainability* **16,** 340 (2024). | Participant　 criteria |
| 3 | Kim, E.-K., Yoon, S., Jung, S. U. & Kweon, S. J. Optimizing urban park locations with addressing environmental justice in park access and utilization by using dynamic demographic features derived from mobile phone data. *Urban For. Urban Green.* **99,** 128444 (2024). | Participant　 criteria |
| 4 | Kim, D.-H., Lee, J. & Yoo, S. Neighbourhood environments for a healthy lifestyle among young single-person households experiencing housing poverty in Seoul, South Korea: a spatiotemporal qualitative study protocol. *BMJ Open* **14,** e077234 (2024). | Participant　 criteria |
| 5 | Bonança, G. M. et al. EEG alpha and theta time-frequency structure during a written mathematical task. *Med. Biol. Eng. Comput.* **62,** 1869–1885 (2024). | Participant　 criteria |
| 6 | Hard, S. A. A. A., Shivakumar, H. N. & Redhwan, M. A. M. Development and optimization of in-situ gel containing chitosan nanoparticles for possible nose-to-brain delivery of vinpocetine. *Int. J. Biol. Macromol.* **253,** 127217 (2023). | Participant　 criteria |

List 2: Reasons for Exclusion Based on Intervention Criteria (N=6).

| No | Article | Reason for exclusion |
| --- | --- | --- |
| 1 | Brailovskaia, J. et al. Less smartphone and more physical activity for a better work satisfaction, motivation, work-life balance, and mental health: An experimental intervention study. *Acta Psychol. (Amsterdam)* **250,** 104494 (2024). | Intervention criteria |
| 2 | Mohapatra, P. et al. Wearable network for multilevel physical fatigue prediction in manufacturing workers. *PNAS Nexus* **3**, 421 (2024). | Intervention criteria |
| 3 | Wei, B. *et al.* Construction site hazard identification and worker adverse reaction monitoring using electroencephalograms: A review. *Buildings* **14,** 180 (2024). | Intervention criteria |
| 4 | Al-Khiami, M. I., Lindhard, S. M. & Wandahl, S. Integrating exoskeletons in the construction sector: a systematic review of empirical evaluation tools and future directions. *Eng. Constr. Archit. Manag.* **33,** 2364–2399 (2026). | Intervention criteria |
| 5 | Görsch, C., Seppänen, O., Peltokorpi, A. & Lavikka, R. Unlocking productivity: revealing waste and hidden disturbances impacting MEP workers. *J. Constr. Eng. Manag.* **150,** 04024108 (2024) | Intervention criteria |
| 6 | Powell, J., Conlon, J. A., Chivers, P. & Cripps, A. J. Exercising smart: exploring the impact of a university staff fitness program on employee productivity and cognition. *J. Occup. Environ. Med.* **67,** e835–e843 (2025). | Intervention criteria |

List 3: Reasons for Exclusion Based on Outcome Criteria (N=15).

| No | Article | Reason for exclusion |
| --- | --- | --- |
| 1 | Rinne, J. et al. An intervention to address nurse educators' occupational well-being: a process evaluation. *Nurse Educ. Today* **138,** 106219 (2024). | Outcome criteria |
| 2 | Edwardson, C. et al. Participant and workplace champion experiences of an intervention designed to reduce sitting time in desk-based workers: SMART work & life. *Int. J. Behav. Nutr. Phys. Act.* **20,** 142 (2023). | Outcome criteria |
| 3 | Lee, M. J., Wang, C. J. & Chang, J. H. Effectiveness of an ergonomic training with exercise program for work-related musculoskeletal disorders among hemodialysis nurses: a pilot randomized control trial. *J. Safety Res.* **91,** 481–491 (2024). | Outcome criteria |
| 4 | Howard-Wilson, S. et al. Efficacy of a multimodal digital behavior change intervention on lifestyle behavior, cardiometabolic biomarkers, and medical expenditure: protocol for a randomized controlled trial. *JMIR Res. Protoc.* **13,** e50378 (2024). | Outcome criteria |
| 5 | Akkarakittichoke, N., Jensen, M. P., Sitthipornvorakul, E. & Janwantanakul, P. Mediators and moderators of a walking intervention to prevent neck pain among high-risk office workers: a secondary analysis of a randomized controlled trial. *Musculoskelet. Sci. Pract.* **71,** 102939 (2024). | Outcome criteria |
| 6 | Indra, B., Palmasutra, V. & Setyawan, F. A. Effectiveness of digital interventions in reducing occupational stress: a systematic review. *Port J. Public Health* **42,** 252–265 (2024). | Outcome criteria |
| 7 | Peterson, N. E., Thomas, M., Hunsaker, S., Stewart, T. & Collett, C. J. mHealth gratitude exercise mindfulness app for resiliency among neonatal intensive care unit staff: three-arm pretest-posttest interventional study. *JMIR Nurs.* **7,** e54561 (2024). | Outcome criteria |
| 8 | Rozga, M., Jones, K., Robinson, J. & Yahiro, A. Nutrition and physical activity interventions for the general population with and without cardiometabolic risk: a scoping review. *Public Health Nutr.* **24,** 4718–4736 (2021). | Outcome criteria |
| 9 | Chen, M., Sparkes, V. & Sheeran, L. Systematic review of digital health interventions to support self-management of low back pain in the workplace. *Digit Health* **11,** 20552076251336281 (2025). | Outcome criteria |
| 10 | Alòs, F. et al. Impact of a primary care-based mobile health intervention to 'sit less and move more' on HbA1c, blood pressure, and other clinical outcomes in office employees with type 2 diabetes: a randomized controlled trial. *Prim. Care Diabetes* **19,** 434–445 (2025). | Outcome criteria |
| 11 | Chandrasekaran, B. & Rao, C. R. Mobile interventions for reducing sedentary behavior and promoting physical activity among office workers: bibliometric study. *Health Educ. Behav.* **53,** 190–200 (2026). | Outcome criteria |
| 12 | Jia, Y. et al. Effects of group communication norms on daily steps in a team-based financial incentive mobile phone intervention in Shanghai, China. *Int. J. Behav. Nutr. Phys. Act.* **22,** 9 (2025). | Outcome criteria |
| 13 | Keyaerts, S., Szymanski, M., Godderis, L., Vanden Abeele, V. & Daenen, L. Evaluating the impact on pain perceptions, pain intensity, and physical activity of a mobile app to empower employees with musculoskeletal pain: mixed methods pilot study. *JMIR Form. Res.* **9,** e67886 (2025). | Outcome criteria |
| 14 | Chandrasekaran, B. et al. Development, implementation and evaluation of a smartphone application aimed to reduce sedentary time and increase physical activity among Indian sedentary office workers: findings from SMART-STEP trial. *BMC Public Health* **25,** 1806 (2025). | Outcome criteria |
| 15 | Chandrasekaran, B., Rao, C. R., Pesola, A. J. & Arumugam, A. Effectiveness of technology-assisted and self-directed interventions to sit less and move more among Indian desk-based office workers: a three-arm cluster randomised controlled trial (SMART-STEP trial). *Appl. Ergon.* **127,** 104528 (2025). | Outcome criteria |

List 4: Reasons for Exclusion Based on Not original article (N=4).

| No | Article | Reason 　　for exclusion |
| --- | --- | --- |
| 1 | Blake, H., Chaplin, W. J. & Gupta, A. The effectiveness of digital interventions for self-management of chronic pain in employment settings: a systematic review. *Br. Med. Bull.* **151,** 36–48 (2024). | Not 　original article |
| 2 | Boucher VG, et al. Effects of 12 weeks of app-based exercise on depressive symptoms of healthcare workers: a randomized controlled trial. *Psychosom. Med.* **85,** A171-A172 (2023). | Not 　original article |
| 3 | Vandelanotte, C. et al*.* MoveMentor-examining the effectiveness of a machine learning and app-based digital assistant to increase physical activity in adults: protocol for a randomised controlled trial. *Trials.* **26,** 233 (2025). | Not 　original article |
| 4 | Can digital health counseling reduce sick leave? ISRCTN15074822. World Health Organization International Clinical Trials Registry Platform (ICTRP) (2025). | Not 　original article |
