# Supplementary material for: Effectiveness of mHealth Interventions Aimed at Promoting Physical Activity and Reducing Sedentary Behavior on Work-Related Outcomes Among Workers: Systematic Review
Source: J Med Internet Res. 2026 May 7;28:e80540. doi: 10.2196/80540 (PMC13152228; doi:10.2196/80540)
Supplement: Multimedia Appendix 4 [file jmir-v28-e80540-s004.docx]

Multimedia Appendix 4: Synthesis Without Meta-Analysis (SWiM) in Systematic Reviews: Reporting Guidelines Checklist.

The citation for the Synthesis Without Meta-analysis explanation and elaboration article is: Campbell M, McKenzie JE, Sowden A, Katikireddi SV, Brennan SE, Ellis S, Hartmann-Boyce J, Ryan R, Shepperd S, Thomas J, Welch V, Thomson H. Synthesis without meta-analysis (SWiM) in systematic reviews: reporting guideline BMJ 2020;368:l6890 <http://dx.doi.org/10.1136/bmj.l6890>

| **SWiM is intended to complement and be used as an extension to PRISMA** | | | |
| --- | --- | --- | --- |
| **SWiM reporting**  **item** | **Item description** | **Page in manuscript**  **where item is reported** | **Other*** |
| *Methods* | | | |
| **1** Grouping studies for synthesis | 1a) Provide a description of, and rationale for, the groups used in the synthesis (e.g., groupings of populations, interventions, outcomes, study design) | Pages 16-17 |  |
|  | 1b) Detail and provide rationale for any changes made subsequent to the protocol in the groups used  in the synthesis | Page 17 |  |
| **2** Describe the standardised metric and transformation methods used | Describe the standardised metric for each outcome. Explain why the metric(s) was chosen, and describe any methods used to transform the intervention effects, as reported in the study, to the standardised metric, citing any methodological guidance consulted | Pages 15-16 |  |
| **3** Describe the  synthesis methods | Describe and justify the methods used to synthesise the effects for each outcome when it was not possible to undertake a meta-analysis of effect estimates | Pages 14-15 |  |
| **4** Criteria used to prioritise results for summary and synthesis | Where applicable, provide the criteria used, with supporting justification, to select the particular studies, or a particular study, for the main synthesis or to draw conclusions from the synthesis (e.g., based on study design, risk of bias assessments, directness in relation to the review question) | Pages 15-16 |  |

| **SWiM reporting**  **item** | **Item description** | **Page in manuscript**  **where item is reported** | **Other*** |
| --- | --- | --- | --- |
| **5** Investigation of heterogeneity in reported effects | State the method(s) used to examine heterogeneity in reported effects when it was not possible to undertake a meta-analysis of effect estimates and its extensions to investigate heterogeneity | Pages 15-16 |  |
| **6** Certainty of evidence | Describe the methods used to assess certainty of the synthesis findings | Pages 14-15 |  |
| **7** Data presentation methods | Describe the graphical and tabular methods used to present the effects (e.g., tables, forest plots, harvest plots).  Specify key study characteristics (e.g., study design, risk of bias) used to order the studies, in the text and any tables or graphs, clearly referencing the studies included | Pages15,17 |  |
| *Results* | | | |
| **8** Reporting results | For each comparison and outcome, provide a description of the synthesised findings, and the certainty of the findings. Describe the result in language that is consistent with the question the synthesis addresses, and indicate which studies contribute to the synthesis | Pages 24-38 |  |
| *Discussion* |  |  |  |
| **9** Limitations of the synthesis | Report the limitations of the synthesis methods used and/or the groupings used in the synthesis, and how these affect the conclusions that can be drawn in relation to the original review question | Page 51 |  |

PRISMA=Preferred Reporting Items for Systematic Reviews and Meta-Analyses.

*If the information is not provided in the systematic review, give details of where this information is available (e.g., protocol, other published papers (provide citation details), or website (provide the URL)).
